# Comparative earthquake sensitivity of straight and triply helical fibers in surface distributed acoustic sensing

**DOI:** 10.1038/s41598-026-54482-4

**Published:** 2026-05-27

**Authors:** Shogo Masaya, Ryohei Naruse, Yuki Kobayashi

**Affiliations:** INPEX Corporation, Akasaka Biz Tower 5-3-1 Akasaka, Minato-ku, Tokyo 107-6332 Japan

**Keywords:** Optical techniques, Geophysics, Seismology

## Abstract

Although distributed acoustic sensing (DAS) using optical fiber cables offers superior spatial and temporal resolution, surface-deployed DAS still faces a significant challenge: straight fiber (SF) cables predominantly record vibrations parallel to cable orientation, limiting the accurate vertical components. Helically wound fiber (HWF) configurations have been proposed to address this limitation of SF and capture multi-directional seismic waves. However, because we cannot perfectly distinguish the seismic signals from the noises in field data, evaluating the comparative sensitivity to large-scale vibrations, such as seismic waves, acquired by SF and HWF is challenging. This study aims to precisely access the sensitivity of HWFs and SFs in surface-deployed DAS measurements for seismic waves excited by earthquake events, which likely propagate vertically. We present a novel approach to analyze the sensitivity of SFs and HWFs by deploying three SFs and triply helically wound fibers, which comprise three HWFs with identical specifications within a single cable. Our results show that the sensitivity ratio of HWF to SF consistently maintains a value of about 0.6 in the conditions of this study. This finding indicates a discrepancy with existing theories on HWF sensitivity that depend on its wrapping angle.

**Supplementary Information:**

The online version contains supplementary material available at 10.1038/s41598-026-54482-4.

## Introduction

In recent years, distributed acoustic sensing (DAS) utilizing an optical fiber cable has emerged as a significant sensing technology with various applications. DAS measures dynamic strain or strain rate along the optical fiber cable. Analysis of data obtained from DAS measurements enables direct derivation of information regarding the phase of vibrations. Amplitude estimation can be attempted by combining phase data with additional information and methodologies. Hence, DAS with an optical fiber cable can record vibrations with higher spatial and temporal resolution than conventional geophones and hydrophones. DAS has been widely employed for subsurface geophysical monitoring by installing optical fiber cables in boreholes (e.g.,^1^). The applications of DAS are currently being expanded. For example, the number of studies reporting DAS measurements deployed at the surface (e.g.,^2–7^) and seafloor (e.g.,^8–10^) is increasing. Many studies employed existing optical fibers deployed at the surface or seafloor, so-called dark fibers for telecommunication, on DAS measurements (e.g.,^8,11^).

**Fig. 1:**
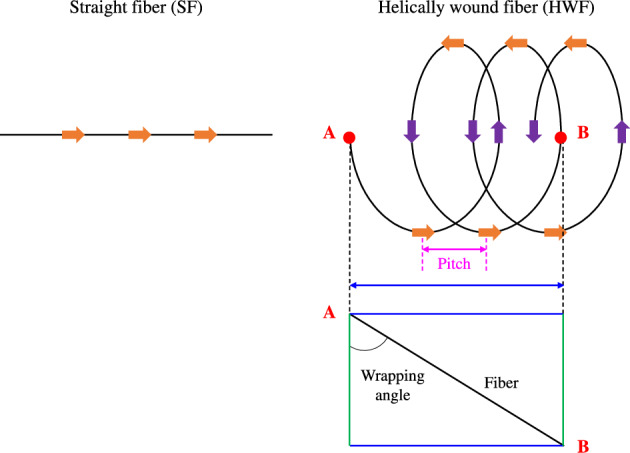
Comparison of schematic images between straight and helically wound fiber. The arrows show that the fiber sensitivity is predominantly along the fiber axis. The definitions of the wrapping angle and pitch of the HWF are illustrated.

However, surface-deployed DAS measurements face a significant challenge arising from the inherent limitation of straight fiber (SF) cable DAS systems, which predominantly record vibration components parallel to the cable orientation. Consequently, accurate data acquisition of vertical vibration components, such as reflected P-waves in active seismic surveys, remains problematic in surface-deployed DAS (Fig. 1). Alternative configurations utilizing helically wound fiber (HWF) have been proposed to address this limitation^2^. HWF aims to enhance the capacity of DAS technology to capture multi-directional vibrations, potentially overcoming the directional sensitivity constraints associated with SF cable deployments (Fig. 1).

Although a couple of studies^6,7^ have reported results from surface DAS measurements using SF and HWF in active seismic acquisition, evaluating the comparative sensitivity of seismic waves acquired by SF and HWF is challenging. This is because we cannot perfectly distinguish the seismic signals from the noises in measured field data. Incorporating geophones into the DAS measurements does not completely resolve this issue, as particle velocity acquired by geophones differs from the strain or strain rate obtained by DAS with optical fibers. Consequently, conversion between particle velocity and strain (rate) is required. Empirical relations are often used to deal with this conversion (e.g.,^12^). Additionally, DAS has particular measurement principles, parameters (e.g., gauge length), and optical noises depending on each type of interrogator. Therefore, the signals of the seismic response recorded by DAS cannot be analytically derived from the seismic response measured by the geophones, and vice versa. Hasani and Drijkoningen (2023) conducted comparative measurements using surface-deployed DAS with SF and HWF. They compared the field data with the synthetic data simulated by forward modeling to analyze the measured DAS data^7^. However, this approach also has the limitation of assuming a plausible subsurface model to run forward modeling and an interrogator model, which might be a black box for interrogator users.

This study presents a novel comparative sensitivity analysis of SF and HWF in surface-deployed DAS by focusing on large-scale vibrations driven by natural earthquakes in DAS data acquired by SFs and triply helically wound fibers (THWFs), which have three HWFs with the same specifications in a single cable. We analyze seismic responses of natural earthquakes for two reasons. One reason is that the relation between the sensitivity of fibers and the magnitude of vibrations can be investigated by utilizing the feature that the energy of seismic waves caused by natural earthquakes attenuates over time. Another reason is that the seismic waves derived from natural earthquakes could be more suitable for the sensitivity analysis of fibers than active seismic waves, which include reflected waves, refracted waves, and surface waves with different propagation angles. To date, no studies have reported on the analysis of natural earthquakes using data from surface-deployed DAS with HWF. Our research provides a novel earthquake analysis by introducing THWFs, thereby improving the accuracy of comparative seismic sensitivity between SF and HWF. By calculating the correlation coefficients of frequency components in the seismic responses measured across these multiple fibers, we quantitatively evaluate whether the components of DAS data exhibit reproducibility across different fiber conditions. Accurate seismic sensitivity of DAS data acquired by SF and HWF can be examined by combining this correlation coefficient analysis with sensitivity analysis.

## Review of difference between SF and HWF

Here, we briefly review an empirical assumption and existing theory on the difference between SF and HWF in DAS before explaining our study. Although SF is commonly used in DAS measurements, a limitation of SF is that the sensitivity to vibrations with the direction of the cable axis is dominant (Fig. 1). Hornman et al. proposed HWF to address this limitation of SF^2^. HWF has the potential to provide sensitivity to not only the direction of the cable axis but also the perpendicular direction of the cable axis by forming the helical shape of fibers (Fig. 1). Several studies^7,13,14^ assume that the fiber longitudinal strain $e_{zz}^{(f)}$ recorded by DAS with HWF is described by

1 $$\begin{aligned} e_{zz}^{(f)} = e_{||}^{(c)} \sin ^2 \alpha + \langle e_{rr}^{(c)} \rangle \cos ^2 \alpha , \end{aligned}$$

where *α* is the wrapping angle of HWF (see Fig. 1).

$e_{||}^{(c)}$ and $\langle e_{rr}^{(c)} \rangle$ represent the cable strain along its axis and the cable radial strain averaged over the cable circumference, respectively. Note that HWF with *α = 90*°is SF. Recent studies^6,7^ report the results of surface-deployed DAS measurements using HWFs with different wrapping angles for onshore active seismic acquisition. However, such studies seem not to conclude whether equation (1) matches the result of the field measurements. One of the reasons would be that $\langle e_{rr}^{(c)} \rangle$ cannot be directly measured, while we can assume that $e_{||}^{(c)}$ is the response observed by DAS with SF. Even though we measure the vertical particle-velocity component by geophones, we still need the assumptions to convert from the vertical particle-velocity component to the vertical strain of $\langle e_{rr}^{(c)} \rangle$. For example, several authors (e.g.,^12^) use the following relationship between the DAS strain ($e_{||}^{(c)}$) measured by SF and the component of particle velocity along the fiber axis ($V_{||}$).

2 $$\begin{aligned} e_{||}^{(c)} (x, t) = \int \frac{V_{||} (x + \frac{l}{2}, t) - V_{||} (x - \frac{l}{2}, t)}{l} dt, \end{aligned}$$

where *x* and *l* represent the position coordinate along the fiber axis and the gauge length used in DAS interrogators, respectively.

Based on a theoretical study on HWF^13^, we consider what kind of data is suitable for evaluating comparative sensitivity of SF and HWF. Kuvshinov (2016) formulated the interaction of HWF with plane seismic waves^13^. He derived the following longitudinal fiber strain for a P-wave under the condition that the boundary of a perfectly coupled cable moves together with the surrounding medium.

3 $$\begin{aligned} e^{(p,coupled)}_{zz} = \cos ^2 \theta \sin ^2 \alpha + \frac{\lambda + 2N - (\lambda _c + 2N)\cos ^2 \theta }{2(\lambda _c + N_c + N)}\cos ^2 \alpha , \end{aligned}$$

where *λ =H-2N*, $N=\rho c_s^2$, $\lambda _c=H_c-2N_c$, $N_c=\frac{E_c}{2(1+\nu _c)}$, $H=\rho c_p^2$, and $H_c=\frac{E_c (1-\nu _c)}{(1+\nu _c)(1-2\nu _c)}$. Here, *ρ*, $c_p$, $c_s$, *E*, and *ν* represent density, P-wave velocity, S-wave velocity, the Young’s modulus, and the Poisson ratio, respectively. The material constants written with the index *c* and without an index refer to the cable and the outer medium, respectively. *θ = 0*°  and *θ = 90*°  represent horizontal and vertical propagation of waves in surface DAS measurements, respectively.

**Fig. 2:**
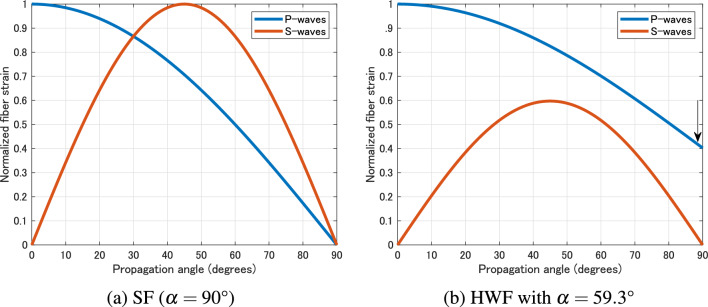
Strain response of P-waves and S-waves for different propagation angles *θ*. Parameters used: density *ρ =1600* kg/m$^3$, cable density $\rho _c=1200$ kg/m$^3$, P-wave velocity $c_p=1500$ m/s, S-wave velocity $c_s=270$ m/s, cable Young’s modulus $E_c=0.8$ GPa, and cable Poisson’s ratio $\nu _c=0.35$. These parameters are partially based on those described in Fig. 3 of Kuvshinov^13^.

Using equation (3), we calculate the longitudinal fiber strain for P-waves and S-waves recorded by SF (*α = 0*°) and HWF with *α = 59.3*°  (Fig. 2), which is a wrapping angle used in our data acquisition. This result indicates a significant difference in sensitivity between HWF and SF for P-waves propagating with the vertical direction (*θ =90*°  in Fig. 2). While SF has no sensitivity to the vertical P-waves, HWF has a particular sensitivity to the vertical P-waves. Therefore, when the propagation of P-waves generated by earthquake events is measured at the surface from a vertical direction, it would be possible to demonstrate the difference in sensitivity between HWF and SF.

However, it should be noted that seismic waves generated by earthquakes are not necessarily guaranteed to propagate from the vertical direction; their propagation is influenced by factors such as the spatial relationship between the source and receivers and the subsurface structure, particularly the shallow velocity structure. Nevertheless, compared to waves originating from active sources, including reflected waves, refracted waves, and surface waves, the seismic waves caused by natural earthquakes are more likely to propagate from the vertical direction to the surface. This argument is based on the applicability of ray theory founded on Snell’s law, as waves in the far field can be described in geometrical optics^15^. According to Snell’s law, waves tend to propagate more vertically as they propagate deeper, and because P-wave velocity increases with depth, the direction of wave propagation approaches vertically under the constant ray parameter condition. Consequently, when P-waves from the far field reach the surface, the incidence angle ($90^{\circ } - \theta$) becomes small, resulting in nearly vertical incidence. This indicates that P-waves from distant sources tend to enter the ground surface almost vertically.

For example, in a study by Park and Ishii, an analysis, including the calculation of incidence angles, was performed on specific seismic events^16^. They used data from the Hi-net network operated by the National Research Institute for Earth Science and Disaster Resilience (NIED) for earthquakes that occurred between January 2004 and April 2016, targeting those with a moment magnitude ($M_w$) *\ge* 6.0 and a focal depth greater than 60 km. While acknowledging that the incidence angle varies with each earthquake and station, they employed a representative P-wave incidence angle of 11.6°  for their simulations. Accordingly, this study operates on the assumption that seismic waves generally exhibit an incidence angle near zero degrees. Another advantage of utilizing natural earthquakes is that their energy attenuates over time, although distinguishing P-wave and S-wave components in attenuated waveforms remains challenging. This characteristic enables us to analyze DAS sensitivity across various vibration magnitudes generated by natural seismic events.

## Data acquisition

The details of our DAS data acquisition using SFs and HWFs are described in this section.

### Triply helically wound fibers (THWFs)

**Fig. 3:**
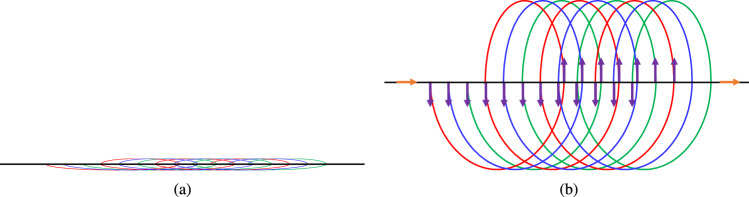
Our used cable with THWFs and an SF. (**a**) The fiber geometry shown at a realistic 1:1 scale, illustrating its high aspect ratio. (**b**) An exaggerated view with the axial scale compressed to clearly depict the distinctive helical features of the THWF and SF structures. The arrows indicate that the fiber sensitivity is predominantly along the fiber axis. Note that the aspect ratio has been altered from a realistic scale in order to clearly depict the distinctive features of the THWF’s structure.

As mentioned, this study employed a novel fiber cable including an SF and THWFs. As illustrated in Fig. 3, THWFs consist of three HWFs designed to remain equidistant from one another throughout a single cable. Each HWF has identical specifications, including the wrapping angle, diameter, and pitch, aside from the locations in a cable. The THWFs were produced by designing *α = 59.3*° , the diameter of 20.0 mm, and the pitch of 100 mm in each HWF. The benefit of employing this cable is that four distinct DAS datasets-three from HWFs and one from SF-can be acquired and compared under identical conditions within the same cable.

### Acquisition geometry and specifications

**Fig. 4:**
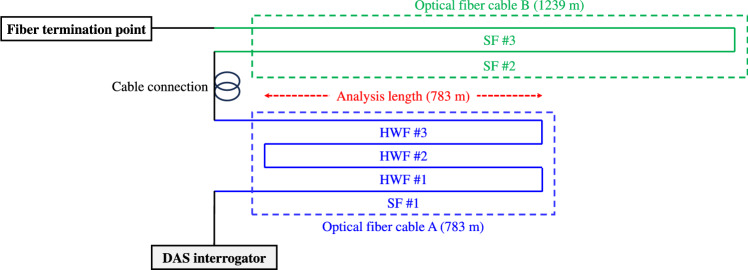
Geometry of DAS measurements with optical fiber cables. While the total buried lengths of optical fiber cable A and optical fiber cable B differ, they are buried in an identical location, with 783 m of overlap. Note that the optical fiber cable A consists of THWFs and SF, as illustrated in Fig. 3.

**Fig. 5:**
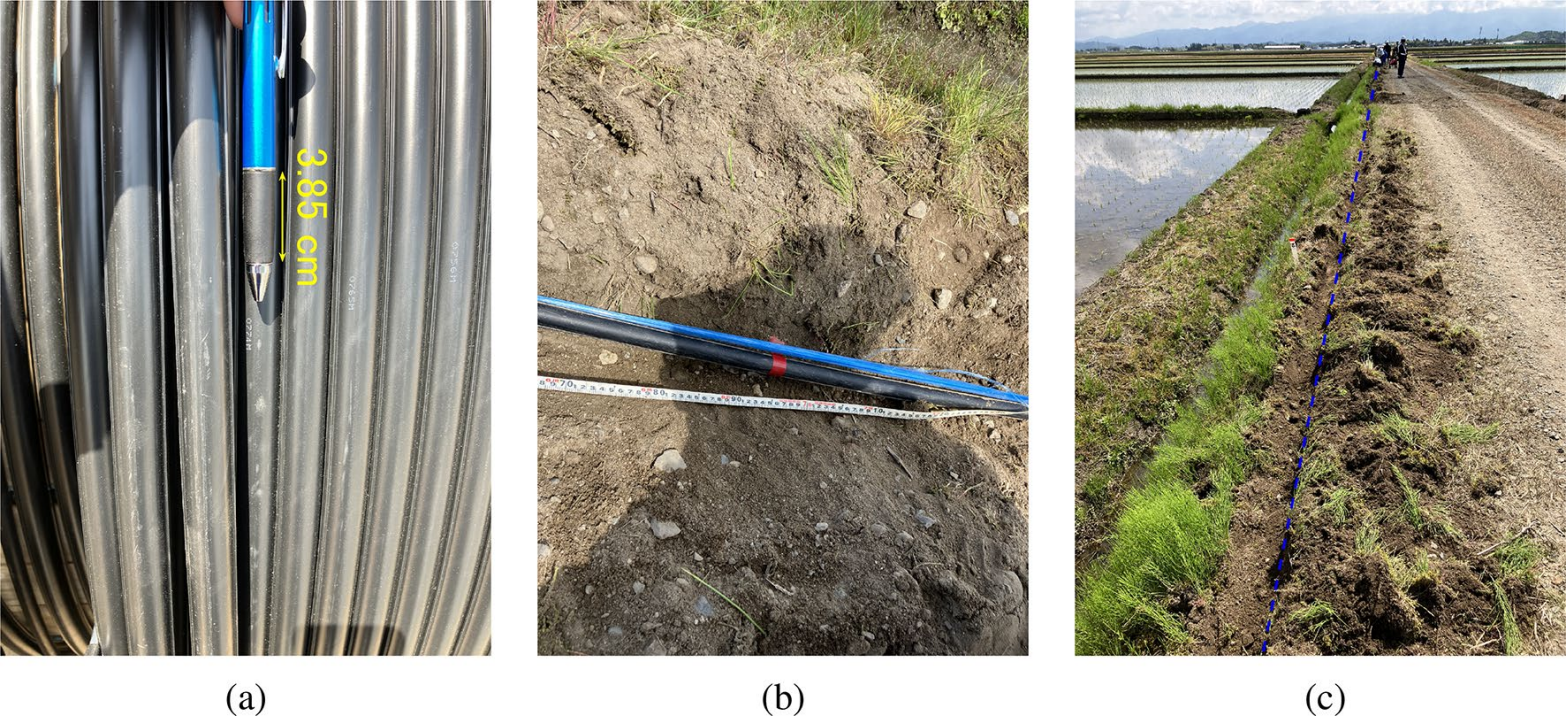
Pictures of cable deployment. (**a**) Pre-deployment optical fiber cable A. Note that the optical fiber cable A includes THWFs and an SF illustrated in Fig. 3. (**b**) Comparison between optical fiber cable A (black) and B (blue). The cable B is wrapped in blue polypropylene tape to facilitate the operation. (**c**) The ground surface above where the optical fiber cables A and B were buried. The cables were deployed under the blue dot line.

Field data was acquired in Niigata prefecture, onshore Japan, from May to June 2023. Figure 4 represents a schematic geometry of two optical fiber cables deployed at a rice paddy area (Fig. 5). While one cable deployed with a total length of 783 m consists of THWFs and one SF, another cable deployed with a total length of 1239 m includes two SFs (Fig. 4). In this paper, we call the former optical fiber cable A and the latter optical fiber cable B. The diameter and jacket material of the optical fiber cable A are 3.8 cm and black FRPE (Flame Retardant Polyethylene), respectively. The buffering between the fiber and jacket in cable A is a tight-buffered construction including an armored tension member. The single-mode fiber is a bend-resistant type (quartz fiber, BIS-B). On the other hand, cable B has a size of 4.3 mm *×* 1.7 mm and includes two tight-buffered SFs. All these fibers are connected end-to-end to conduct DAS measurements with a single interrogator. While the operation of fiber loop-backs within each fiber cable was performed by a cable manufacturer, the connection operation between the two fiber cables was carried out on-site. This paper focuses on the part of the 783 m overlapped by these two cables. These two cables were buried at a shallow level between 10-20 cm in the same line. Our data analysis focuses on the overlapped length of 783 m. Because each wrapping angle of HWF in the THWFs is *α = 59.3*° , the analytical length of the HWF corresponding to the cable length of 783 m is 911 m (*= 783 / \sin 59.3*° ). The produced THWFs have three HWFs with lengths of 908 m, 911 m, and 912 m, respectively, which indicates that the error caused by the cable production is less than 0.1%.

We used a DAS interrogator (NBX-S4000 produced by Neubrex Co., Ltd.) based on optical frequency domain reflectometry in this field test. DAS data was recorded as a strain rate by the parameters of interrogation rate of 1000 Hz, time sampling interval of 1.00 ms, spatial resolution of 1.00 m, gauge length of 0.21 m, and spatial sampling interval of 0.41 m. Utilization of short gauge lengths (e.g., 0.21 m) in DAS presents both advantages and disadvantages. The magnitude of noise originating from the interrogator system remains independent of gauge length; consequently, reducing the gauge length diminishes the measurable signal amplitude, resulting in degradation of the signal-to-noise ratio (e.g.,^17^). Conversely, when monitoring strong motions, such as large-scale seismic events, via DAS, the probability of cycle skipping increases proportionally with the magnitude of temporal amplitude variations (e.g.,^18^). Using shorter gauge lengths and time sampling rates is advantageous to mitigate this issue. According to the manufacturer of the interrogator, the strain rate is output as the median value between the points before and after the gauge length of each sampling point. The manufacturer suggests that calculating the moving average of these output values makes them comparable to the output of other interrogators.

## Data analysis

Here, we state the analysis of the data acquired in this study. The data analysis includes a workflow for data processing and the processing results for two earthquakes.

### Processing workflow

**Fig. 6:**
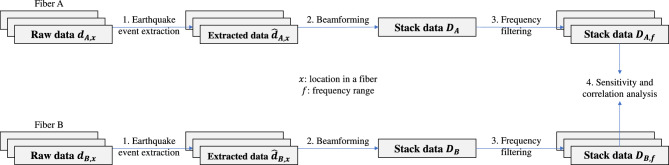
Processing workflow for DAS data acquired from two independently selected optical fibers. Here, we designate these arbitrary fibers as fiber A and fiber B. Note that this designation is independent of cable A and cable B.

The processing workflow in this study consists of the following four steps (Fig. 6).

**Fig. 7:**
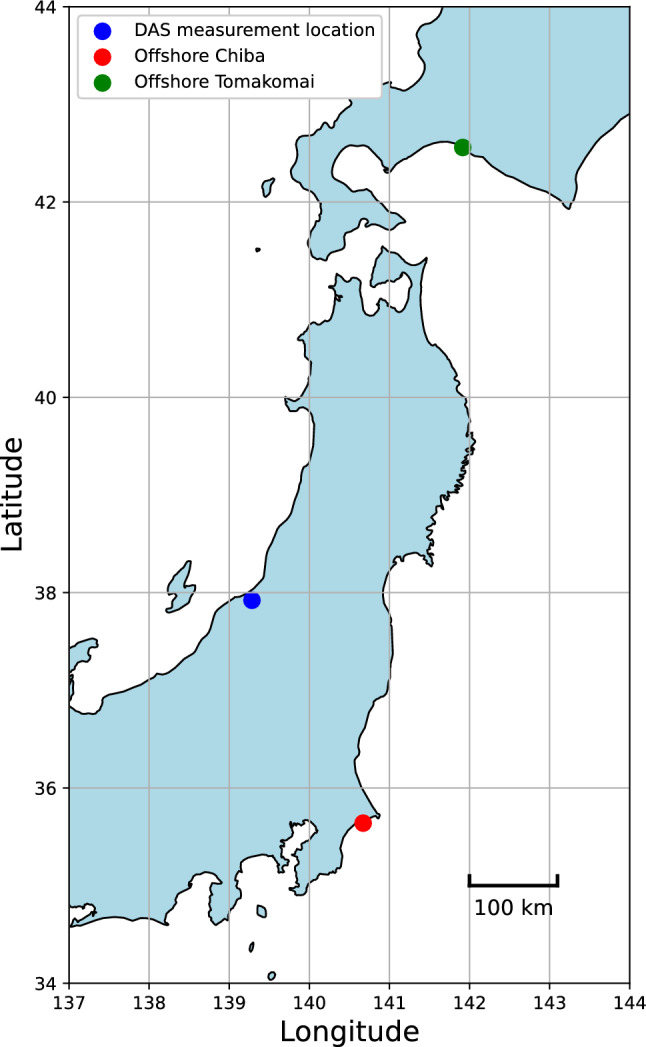
Locations of DAS measurements and earthquake epicenters in Offshore Chiba and Tomakomai, which are shown in Table 1. This figure is generated by modifying https://github.com/datasets/geo-countries.

**Fig. 8:**
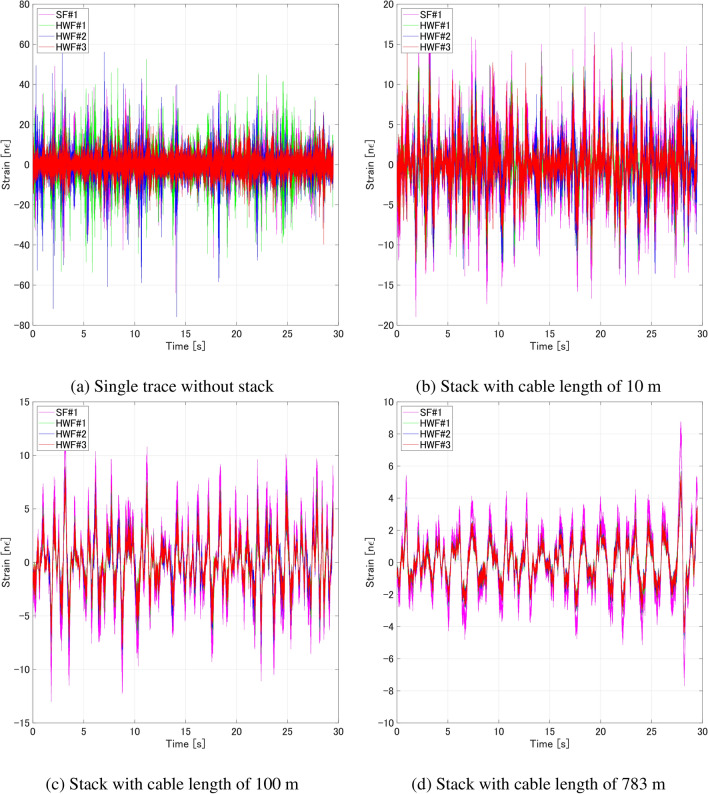
Examples of seismic waveforms with the earthquake event at offshore Chiba after beamforming with varying stack parameters. The 0 s time point in these waveforms corresponds to 19:04:22 JST on May 26, 2023.

**Fig. 9:**
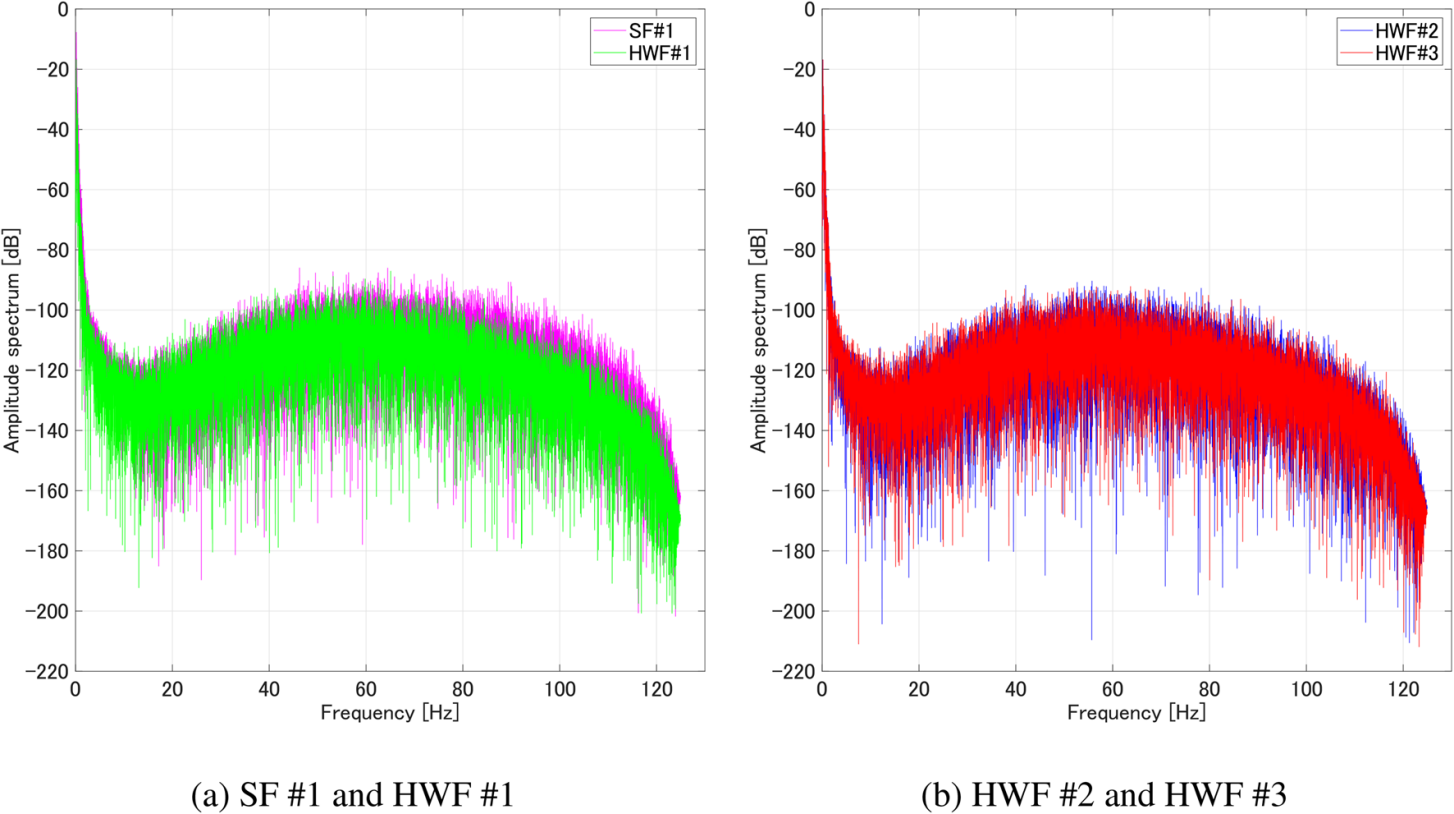
Amplitude spectra of seismic waveforms presented in Fig. 8d.

1. Earthquake event extraction Earthquake events in the acquired DAS data are identified and extracted based on the earthquake information published by the Japan Meteorological Agency (JMA). We pick up two earthquake events with larger magnitude than 6.0 generated around Japan for our analysis (Table 1 and Fig. 7).
2. Beamforming We conduct beamforming for the extracted DAS data, including the earthquake events. In the beamforming process, we pick the first arrivals of the earthquake events in the DAS data and then apply a time shift with a constant velocity to minimize the difference of first arrivals depending on each location of a cable line. Finally, the stack for the time-shifted data is computed at the location of the left edge point of the fiber cable illustrated in Fig. 4. The waveforms calculated after beamforming are changed by depending on the number of samples used in the stack (Fig. 8). The unit of strain in the waveforms is nanostrain (*nε*). Because we utilized a 783 m cable with SFs and HWFs in this study, we elected to use data samples spanning the whole 783 m in our stacking procedure to maximize the cable’s effectiveness in reducing random noise.
3. Frequency filtering Although amplitude spectra can be computed from the stacked waveforms obtained through beamforming (Fig. 9), these spectra alone do not provide sufficient information for a detailed assessment of the fiber’s sensitivity. Hence, we employ frequency filtering to decompose and analyze the waveforms across distinct frequency bands, enabling a more precise evaluation of the fiber’s response characteristics.
4. Sensitivity and correlation analysis Sensitivity and correlation are analyzed from the seismic waveforms across each frequency band. To quantify the relative response of the two fibers, we define the sensitivity ratio $\xi _{AB}$ using the stacked waveforms $D_{A,f}(t)$ and $D_{B,f}(t)$ as follows:
  4 $$\begin{aligned} \xi _{AB} = \frac{\int _t^{t+ \Delta t} |D_{A,f} (t)| dt}{\int _t^{t+ \Delta t} |D_{B,f} (t)| dt}, \end{aligned}$$
  where *Δ t =30* s is used in this study. Furthermore, we calculate the correlation coefficients of frequency components in the seismic responses measured across multiple fibers. This analysis aims to quantitatively evaluate the reproducibility of DAS data measured by different fiber conditions.

**Table 1:** List of earthquakes analyzed in this study.

Date	Time	Locations	Latitude	Longitude	Depth	Distance	Magnitude
–	–	DAS measurement location	37°55.3’ N	139°17.0’ E	Surface	–	–
2023-05-26	19:03:24	Offshore Chiba, Japan	35°38.4’ N	140°40.3’ E	50 km	286 km	6.2
2023-06-11	18:54:45	Offshore Tomakomai, Japan	42°33.5’ N	141°54.9’ E	136 km	578 km	6.2

Event information is based on data published by JMA. The listed distance refers to the separation between the DAS measurement location and the epicenter of each earthquake.

### Simulation of incidence angle using ray tracing

To verify the assumption of near-vertical P-wave incidence angle, we perform numerical ray-tracing simulations for the first arrivals of the two earthquake events. The simulations are conducted using the TauP toolkit^19^ within the ObsPy software package^20^, a standard tool for calculating seismic travel times and ray paths in spherical earth models.

A site-specific 1D P-wave velocity model is constructed by integrating two authoritative datasets:

- Shallow structure (depth < 8 km): We adopt the shallower subsurface structure model from the Japan Seismic Hazard Information Station (J-SHIS) provided by NIED. Specifically, the velocity profile at the DAS measurement site (Mesh Code: 56397103) is utilized to account for the local sedimentary effects.
- Deep structure (depth > 8 km): The deeper crustal and mantle structures are based on the JMA2020A velocity model, the latest standard model used by the Japan Meteorological Agency for earthquake source determination.

**Fig. 10:**
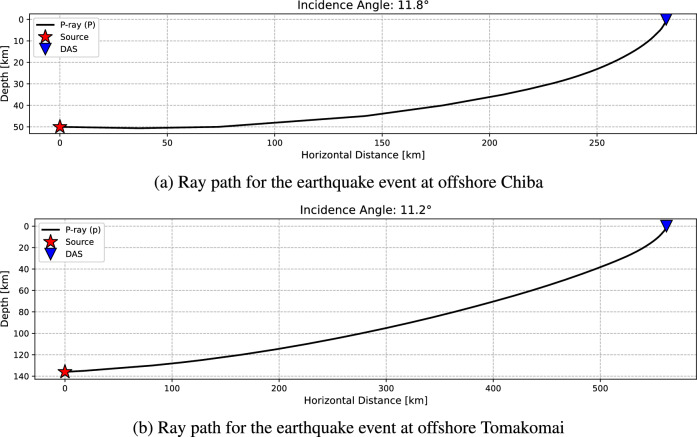
Ray path comparisons of the earthquake events between (a) offshore Chiba and (b) offshore Tomakomai. These results demonstrate that both earthquake events provide near-vertical P-wave incidence, which is ideal for evaluating the directional sensitivity of the fibers.

The ray-tracing results yield first arrival P-wave incidence angles (measured from the vertical) of $11.8^{\circ }$ for the offshore Chiba event and $11.2^{\circ }$ for the offshore Tomakomai event (Fig. 10). These values are in agreement with the representative incidence angle of $11.6^{\circ }$ reported in a previous large-scale study by Park and Ishii (2018) using Hi-net data^16^ published by NIED. Therefore, the assumption that the incidence angles of the earthquake events used in this study are small (i.e., near-vertical) is justified by this simulation. The near-vertical arrival of these P-waves (corresponding to propagation angles $\theta \approx 78^{\circ }$–$79^{\circ }$ in Fig. 2) provides an appropriate condition to demonstrate the sensitivity difference between HWF and SF.

### Earthquake at offshore Chiba, Japan on May 26, 2023

Next, we present an analysis of DAS data that includes the earthquake event off the coast of Chiba, Japan on May 26, 2023. The two seismic events employed in this study (Table 1) share a magnitude of 6.2. However, the distance between the epicenter and the fiber cable differs: the earthquake at offshore Chiba occurred closer to the cable than at offshore Tomakomai (Table 1 and Fig. 7). Therefore, the earthquake at offshore Chiba could exhibit higher energy levels as measured by DAS compared to that at offshore Tomakomai.

**Fig. 11:**
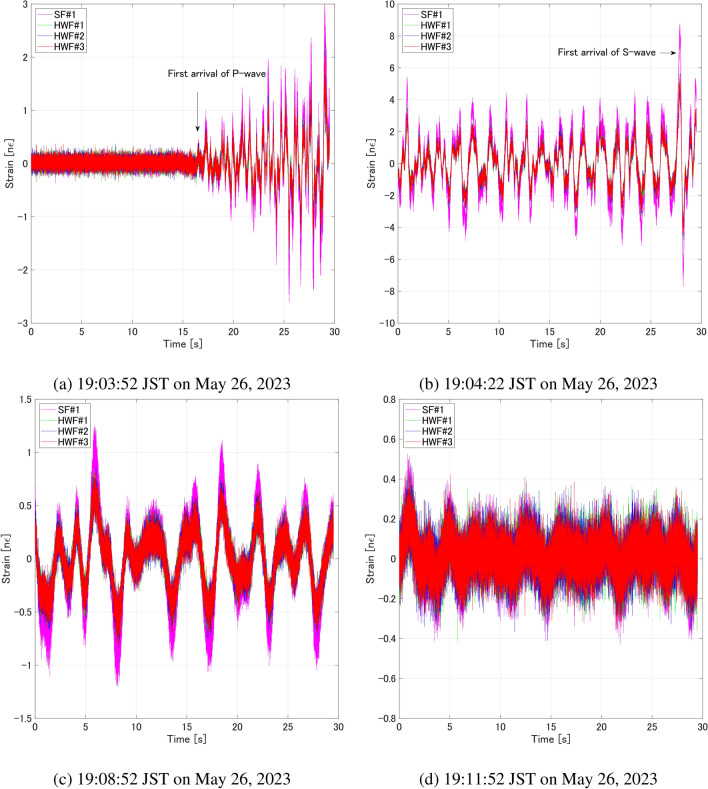
Stacked seismic waveforms before and after the earthquake event at offshore Chiba, Japan on May 26, 2023. The 0-second point on the time axis of these graphs corresponds to the time points (**a**) to (**d**).

The waveforms after beamforming (Fig. 11) show that the first arrival of the P-wave (Fig. 11a), followed by S-waves with larger amplitudes (Fig. 11b), and a gradual amplitude attenuation over time (Fig. 11c, d). Focusing on the differences among fibers, we can observe that for vibrations of a certain magnitude (Fig. 11b, c), the phase exhibits a high degree of similarity regardless of the fiber, with the SF showing relatively higher amplitudes and slight differences among the three HWFs. On the other hand, for smaller magnitude vibrations (Fig. 11d), while the amplitude remains similar across fibers, the phase similarity is less apparent.

**Fig. 12:**
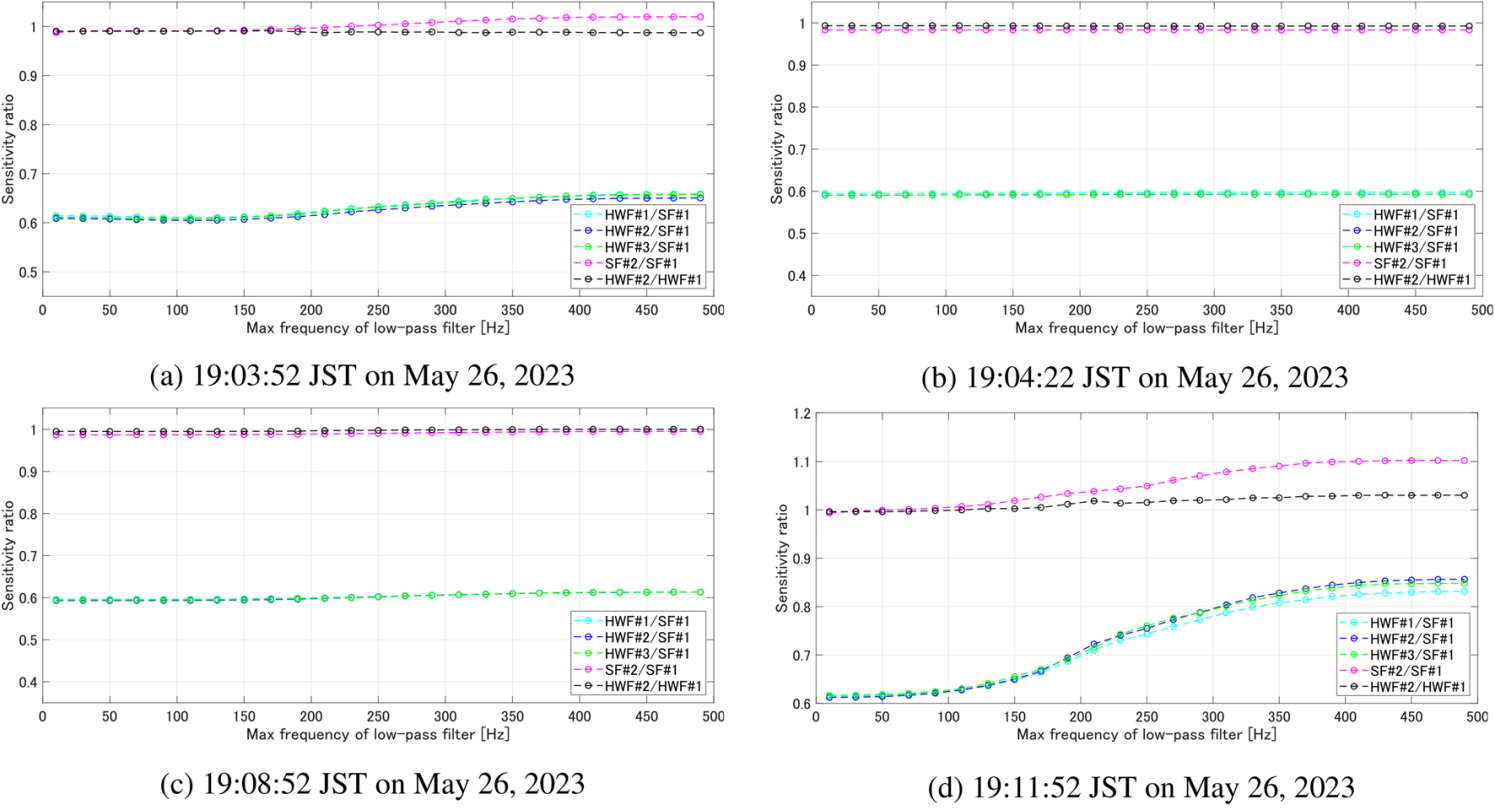
Sensitivity ratio of stacked seismic waveforms before and after the earthquake event at offshore Chiba, Japan on May 26, 2023. These graphs represent the results of analyses using 30-second waveforms from the time points (**a**) to (**d**). The sensitivity ratio of the HWF remains stable at approximately 0.6 for high-magnitude vibrations (**a**-**c**). However, the apparent increase in (**d**) suggests a transition where noise becomes dominant in the high-frequency range.

We apply frequency filters to the stacked seismic waveforms and then calculate sensitivity ratios for combinations of five fiber types across different frequency bands (Fig. 12). For high-magnitude vibrations, the sensitivity ratio remains constant regardless of the maximum frequency of the low-pass filter. The sensitivity of the HWF is found to be approximately 60% of the SF sensitivity (Fig. 12a–c), even for the P-wave (Fig. 12a). Additionally, the sensitivities among SFs and HWFs are nearly identical. However, as seismic energy attenuates, the sensitivity ratio varies depending on the frequency band (Fig. 12d). The sensitivity ratio of HWF to SF increased for stacked seismic waveforms containing high-frequency components. The sensitivity ratio among HWFs remains close to 1, showing no significant change. Under these conditions, SF #2 exhibits a slightly higher sensitivity than SF #1, suggesting that optical fiber cable B may have better coupling conditions than optical fiber cable A. A plausible explanation for these results is that the optical fiber cable A, which incorporates not only SF#1 but also three HWFs, potentially experiences reduced coupling efficiency and sensitivity relative to the cable B, primarily due to its increased cable diameter. While a smaller optical fiber cable diameter reduces the cable contact area with the soil at the near-surface level, it may enhance the contact ratio between the buried cable and the soil. Although this phenomenon may also depend on soil type and burial conditions, the measurements in this study indicate that the contribution of the contact ratio between the buried cable and the near-surface may have been more significant than the contribution of the contact area with the near-surface.

**Fig. 13:**
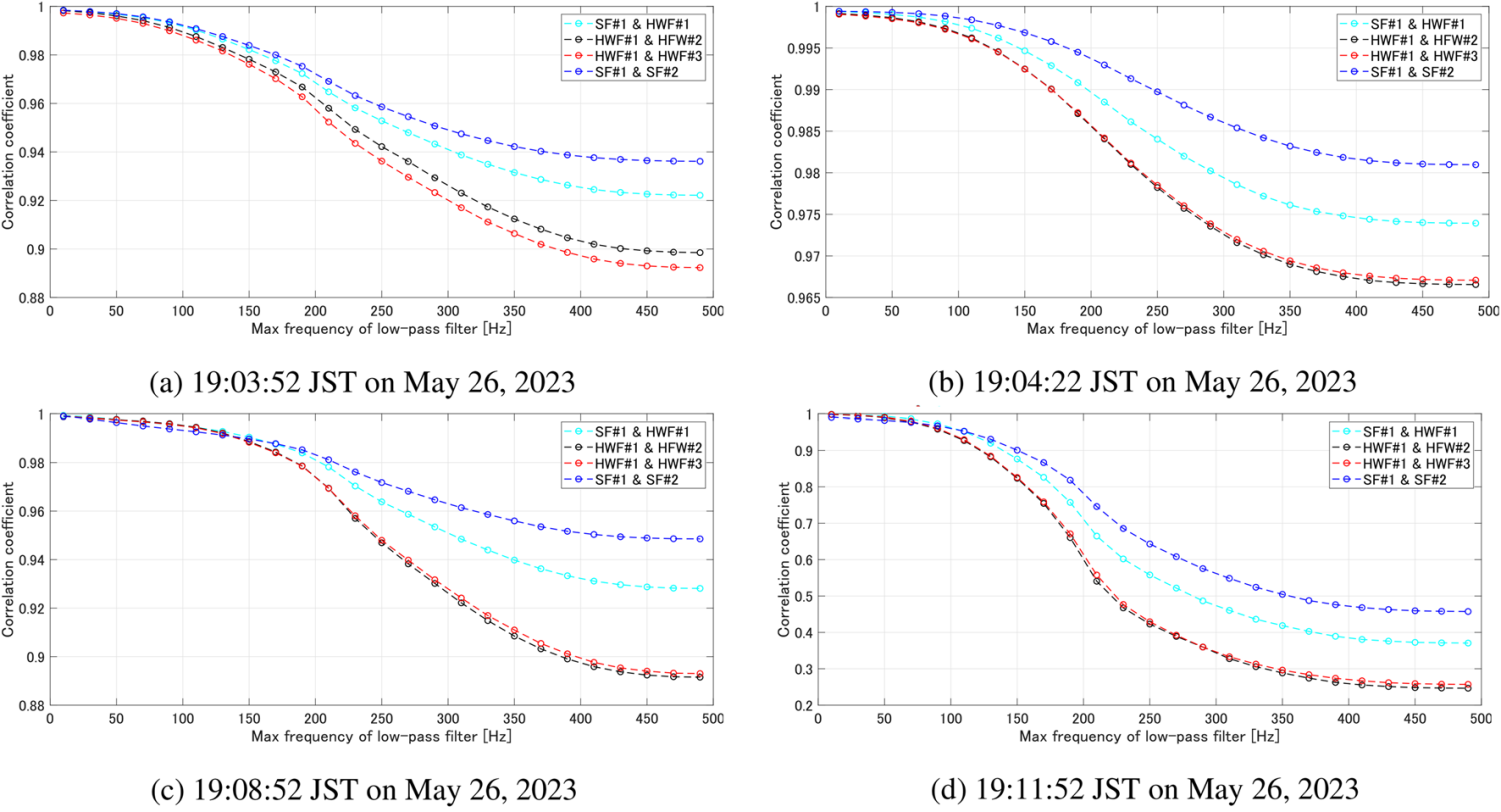
Correlation coefficient of stacked seismic waveforms before and after the earthquake event at offshore Chiba, Japan on May 26, 2023. These graphs represent the results of analyses using 30-second waveforms from the time points (**a**) to (**d**).

Correlation coefficients are calculated for each stacked seismic waveform to examine the frequency-dependent changes in fiber sensitivity for seismic waves with low energy (Fig. 13). A significant decrease in correlation coefficients is observed for stacked seismic waveforms containing high-frequency components in low seismic energy (Fig. 13d). This finding indicates that although the sensitivity ratio of HWF to SF increases, the high-frequency components contributing to this increase are not correlated between HWFs. Therefore, these components likely represent random noise rather than reliable seismic signals.

### Earthquake at offshore Tomakomai, Japan on June 11, 2023

Our previous results indicate that as seismic amplitude decreases, the sensitivity of HWF to high-frequency components increases. However, this frequency range also corresponds to a decrease in correlation coefficients among HWFs, raising concerns about the reliability of these observations. For instance, we cannot rule out the possibility that high-frequency random noise from the interrogator is being measured. To address this uncertainty, we analyze the DAS data with a seismic event at offshore Tomakomai, Japan on June 11, 2023, which has lower energy levels.

Using the same analytical approach as the previous seismic event, we compute the stacked seismic waves (Fig. S1) before and after the seismic event off the coast of Tomakomai and then calculate the sensitivity ratios (Fig. S2) and correlation coefficients (Fig. S3) for the stacked seismic waves. Note that Figs. S1–S9 are shown in the material. The analysis results show that for seismic waves with relatively large energy immediately after the first arrival of the P-wave, the sensitivity ratio of the HWFs to the SFs is approximately 0.6 (Fig. S2b), which is almost the same as the result of the previous seismic event, indicating a reproducible characteristic. Subsequently, this seismic event is observed to be in a state where the seismic wave energy is further attenuated (Fig. S2d). Even in the low-frequency domain (0-50 Hz) at this stage, the sensitivity ratio of the HWF to the SF exceeds 1 (Fig. S2d), and the correlation coefficient is also greater than 0.8 (Fig. S3d), indicating that this phenomenon cannot be interpreted as the influence of random noise. To observe the details of this phenomenon, different low-cut filters are applied to the 30-second stacked seismic waveforms from 19:06:10 JST on June 11, 2023. The filtered waveforms show that in the low-frequency domain (0-50 Hz), the waveforms of the HWFs exhibit a positive baseline shift compared to those of the SF (Fig. S4a, b), though such shift of the waveforms including higher frequencies (50-130 Hz) is not so clear (Fig. S4c, d).

To quantitatively investigate this baseline shift, we define the positive sensitivity ratio, $\xi _{AB}^+$, and the negative sensitivity ratio, $\xi _{AB}^-$, as

5 $$\begin{aligned} \xi _{AB}^+= & \frac{\int _t^{t+ \Delta t} \max \{0, D_{A,f} (t)\} dt}{\int _t^{t+ \Delta t} \max \{0, D_{B,f} (t)\} dt}, \end{aligned}$$

6 $$\begin{aligned} \xi _{AB}^-= & \frac{\int _t^{t+ \Delta t} \min \{0, -D_{A,f} (t)\} dt}{\int _t^{t+ \Delta t} \min \{0, -D_{B,f} (t)\} dt}. \end{aligned}$$

The positive sensitivity ratio represents the sensitivity ratio of the integral value of only the positive values of waveforms, while the negative ratio uses the absolute values of negative values of waveforms. The results show that for seismic waves with large energy, both the positive sensitivity ratio and the negative sensitivity ratio of the HWFs and SF #1 remain at around 0.6 (Fig. S5b, S6b). However, when focusing on seismic waves with smaller energy, in the low-frequency domain (0-50 Hz), the positive sensitivity ratio of the HWFs and SF #1 is 1.8-1.9 (Fig. S5d), while the negative sensitivity ratio is almost zero, indicating no sensitivity (Fig. S6d). For seismic waves with intermediate energy levels, the positive sensitivity ratio of HWFs and SF #1 is slightly higher than the negative sensitivity ratio (Figs. S5a and c, S6a and c), due to the positive baseline shift of these stacked waveforms.

## Discussion

Here, we discuss the validity of the sensitivity ratio of HWF to SF in the above data analysis. When the magnitude of seismic vibrations is large, the sensitivity ratio of HWF to SF remained stable at about 0.6, with no significant variation observed among individual fibers. In the presence of low-magnitude seismic vibrations, the sensitivity ratio of HWF to SF was observed to exceed 1.0 within 0-50 Hz of the seismic waveforms with high correlation coefficients among data measured by different HWFs. To precisely evaluate the detail of the sensitivity ratio, we analyze the mechanism of the positive amplitude baseline phenomenon in seismic waveforms measured by HWFs and then state the baseline correction of the stacked seismic waveforms measured by HWFs for the low-magnitude seismic vibrations and stack number dependency of the sensitivity ratio in beamforming. Finally, we discuss the discrepancy between DAS measurements in this study and previous studies.

### Positive baseline shift of stacked seismic waveforms measured by HWFs

While it is challenging to present a comprehensive mechanism and solid evidence that reasonably explains the positive baseline shift phenomenon observed in HWFs, we describe an interpretative framework. The optical fiber cable A is buried near the surface, resulting in soil deposition above the cable. The external force generated by the weight of this soil and the cable itself can be understood to act on the optical fibers in a nearly constant manner along the gravitational direction. Through simple assumptions, we can estimate the soil and cable weights to compare their respective contributions to the gravitational external force acting upon the optical fibers. For simplicity, we assume the cable is uniformly buried at a depth of 15 cm, with a soil bulk density of 1500 kg/m$^3$. Given that the optical fiber cable A has a diameter of 3.8 cm, the soil volume per meter of cable can be calculated as 1 m *×* 0.038 m *×* 0.15 m = 0.0057 m$^3$, yielding an estimated soil weight per meter of cable = 0.0057 m$^3$ *×* 1500 kg/m$^3$ = 8.55 kg/m. On the other hand, while the authors lack specific information regarding the weight of optical fiber cable A except the fibers, we know that the total weight of 800 m of this cable is approximately 580 kg. Thus, we can approximate the weight per meter as 580 kg / 800 m = 0.725 kg/m. These approximations reveal that the external force with gravitational direction acting on the surface-deployed fibers is predominantly attributable to the soil rather than the cable itself. Consequently, the helical structure of HWFs potentially exhibits directional sensitivity, particularly to forces applied along the gravitational axis (Fig. S7). In contrast, SF does not have a sufficient sensitivity to the gravitational direction, resulting in reduced susceptibility to this external force. This differential sensitivity may be associated with the phenomenon wherein waveforms acquired through HWFs exhibit a slight positive baseline shift compared to those obtained via SFs.

### Baseline correction of stacked seismic waveforms measured by HWFs

We correct the mentioned positive baseline shift of DAS waveform data measured by the HWFs (Fig. S8a) and then calculate the sensitivity ratio of the corrected seismic waveforms. As stated, the sensitivity of HWFs in the low-frequency range (0-50 Hz) of recorded seismic data seems to be larger than that of SF #1 (Fig. S8b). However, due to the positive baseline shift recorded by HWFs, the sensitivity ratio of HWF to SF may be overestimated. To analyze this point, we perform baseline correction by subtracting a constant positive trend, the mean value of the data measured by HWF. After this baseline correction, it becomes evident that HWF sensitivity is lower compared to SF, even for low-frequency waveforms (Fig. S8c, d). Using the corrected waveforms to calculate the sensitivity ratio of HWFs to SF, we find that in the low-frequency range (0-50 Hz) of the seismic data with high correlation coefficients of the data measured by HWFs, the ratio is approximately 0.6, nearly identical to the sensitivity ratio to relatively large vibrations observed. Therefore, it indicates that, regardless of the magnitude of vibrations, these HWFs have approximately 60% sensitivity of SF for the seismic events analyzed in this study. Our study is the first to quantitatively demonstrate that the sensitivity of HWFs to SFs is approximately 60%. This exact value of 60% depends on the specification of the HWFs used in our study, and the sensitivity value would likely vary with different HWF specifications.

### Stack number dependency of sensitivity ratio

Because the stacking process in this analysis is determined by a parameter of fiber cable length, there is a difference in spatial data samples between SF and HWF. The SF #1 length of 783 m corresponds to the HWF #1 length of 908 m, the HWF #2 length of 911 m, and the HWF #3 length of 912 m. Since DAS measurements in this study acquire the data with the spatial sampling interval of 0.41 m in fiber length rather than cable length, the SF of 783 m has 1,909 spatial data samples (= 783/0.41), while the HWF of 908 m has 2,214 spatial data samples (= 908/0.41). Although it is preferable to define the stack using cable length in terms of physical location comparisons, this approach incurs the issue that the number of spatial data samples for HWF and SF is not identical. Therefore, we present the calculated results for the case where the data samples for HWFs are also stacked using 1,909 spatial data samples corresponding to the 783 m fiber length of SF (Fig. S9). Even when considering these results, the sensitivity ratio between HWF and SF remains approximately 0.6, indicating that the difference in the number of stacks between HWF and SF is unlikely to have a crucial impact on the sensitivity ratio. Thus, it is evident that the sensitivity ratio of HWF to SF consistently maintains a stable value of 0.6, regardless of variations in the stacking calculation conditions.

### Discrepancy between DAS measurements in this study and previous studies

Since there is a discrepancy between DAS sensitivity in this study and previous studies that include equation (1) and (3), we discuss the causes that incurred this difference. When HWF with the wrapping angle *α =59.3*°  is applied to equation (1),

7 $$\begin{aligned} e_{zz}^{(f)} = e_{||}^{(c)} \sin ^2 \alpha + \langle e_{rr}^{(c)} \rangle \cos ^2 \alpha = 0.739 e_{||}^{(c)} + 0.261 \langle e_{rr}^{(c)} \rangle . \end{aligned}$$

Hence, we can calculate the relative ratio of HWF to SF for horizontal and vertical weight (Table 2). As discussed, because the sensitivity ratio of HWF to SF consistently maintains a stable value of 0.6, regardless of variations in the stacking calculation conditions, there are quantitative discrepancies between sensitivity weight computed by equation (1) and sensitivity analyzed in this study (Table 2). The actual sensitivity of HWFs also would depend on various factors, including fiber material properties and helical diameter, suggesting a higher degree of complexity than the equations account for. The HWFs utilized in this study have been shown to exhibit approximately 0.6 times the sensitivity of SFs for both P-waves and S-waves. This suggests that contrary to the expectation that HWFs might possess unique directional sensitivities not present in SFs (as illustrated in Fig. 2b), they more likely demonstrate an overall reduction in sensitivity across all directions when compared to SFs (Fig. 2a).

**Table 2:** Comparison between sensitivity weight computed by equation (1) and sensitivity analyzed in this study.

	Theory: horizontal weight	Theory: vertical weight	Experiment
SF	1.000	0.000	
HWF with $\alpha =59.3^\circ$	0.737	0.261	
[SF] / [HWF with $\alpha =59.3^\circ$]	1.000/0.737 = 1.353	0.000/0.261 = 0.000	
This study: [SF] / [HWF with $\alpha =59.3^\circ$ ]			1.0/0.6 = 1.7

## Conclusions

This study investigated the sensitivity of HWFs and SFs in surface-deployed DAS measurements for seismic waves derived from earthquake events, which likely originate from vertical propagation. We used three SFs and THWFs to distinguish seismic signals from noises through correlation coefficient analysis and sensitivity analysis for the DAS data recorded by multiple fibers. Under high-magnitude seismic vibrations, the sensitivity ratio of HWFs with *α =59.3*°  to SF stabilizes at approximately 0.6 in the conditions of this study, as demonstrated consistently across the three HWF samples. In the presence of low-magnitude seismic vibrations, the helical-to-straight ratio can exceed 0.6 in the seismic waves of 0-50 Hz. Here, we observed a phenomenon where the sensitivity of HWFs exhibits a positive baseline shift of the waveforms observed under these conditions. However, we also demonstrated that even for small vibrations, when the positive baseline shift measured by HWFs is subtracted from the stacked seismic waveforms, the sensitivity ratio of HWF to SF approaches approximately 0.6. Therefore, we found that the sensitivity ratio of HWF to SF consistently maintains a stable value of 0.6 for two earthquake events, regardless of variation magnitude. Furthermore, the results suggest that the HWFs used in this study are more likely to exhibit an overall reduction in sensitivity compared to SFs, rather than demonstrating directional sensitivities not present in SFs. This finding indicates a discrepancy with existing HWF theories based on equation (1) that do not account for factors such as fiber material composition or helical diameter in HWF. Finally, a limitation of this study is that the incidence angle is not exactly $0^{\circ }$; instead, it is estimated to be approximately $11^{\circ }$ based on our ray-tracing simulations and the previous study^16^. Moreover, investigating the influence of the interrogator’s gauge length also warrants future study.

## Electronic Supplementary Material

Below is the link to the electronic supplementary material.

Supplementary Material

## Data Availability

The datasets used and/or analysed during the current study available from the corresponding author on reasonable request.

## References

[1] Daley, T. M. et al. Field testing of fiber-optic distributed acoustic sensing (DAS) for subsurface seismic monitoring. *Lead. Edge* **32**, 699–706. 10.1190/tle32060699.1 (2013).

[2] Hornman, K., Kuvshinov, B., Zwartjes, P. & Franzen, A. Field trial of a broadside-sensitive distributed acoustic sensing cable for surface seismic. In *Proceedings of 75th EAGE conference & exhibition*, Tu 04 08, 10.3997/2214-4609.20130383 (2013).

[3] Bakulin, A., Silvestrov, I. & Pevzner, R. Surface seismics with das: An emerging alternative to modern point-sensor acquisition. *Lead. Edge* **39**, 765–844. 10.1190/tle39110808.1 (2020).

[4] Yang, J., Shragge, J. & Jin, G. 4D DAS fiber-coupling effects in freezing near-surface ground conditions. In *Proceedings of 1st international meeting for applied geoscience & energy* 477–482, 10.1190/segam2021-3582508.1 (2021).

[5] Harmon, N. et al. Surface deployment of DAS systems: Coupling strategies and comparisons to geophone data. *Near Surf. Geophys.* **4**, 465–477. 10.1002/nsg.12232 (2022).

[6] Bakulin, A., Alfataierge, E. & Pevzner, R. Evaluating HD weathering surveys and surface seismic with DAS in a sand dune environment. In *Proceedings of 2nd international meeting for applied geoscience & energy* 621–625, 10.1190/image2022-3735875.1 (2022).

[7] Hasani, M. A. & Drijkoningen, G. Experiences with distributed acoustic sensing using both straight and helically wound fibers in surface-deployed cables - A case history in Groningen, The Netherlands. *Geophysics* **88**, B369–B380. 10.1190/geo2022-0769.1 (2023).

[8] Zhan, Z. et al. Optical polarization-based seismic and water wave sensing on transoceanic cables. *Science* **371**, 931–936. 10.1126/science.abe6648 (2021). 33632843 10.1126/science.abe6648

[9] Shinohara, M., Yamada, T., Akuhara, T., Mochizuki, K. & Sakai, S. Performance of seismic observation by distributed acoustic sensing technology using a seafloor cable off Sanriku, Japan. *Front. Mar. Sci.*10.3389/fmars.2022.844506 (2022).

[10] Shen, Z. & Wu, W. Ocean bottom distributed acoustic sensing for oceanic seismicity detection and seismic ocean thermometry. *J. Geophys. Res.: Solid Earth* **139**, e2023JB027799. 10.1029/2023JB027799 (2024).

[11] Ajo-Franklin, J. B. et al. Distributed acoustic sensing using dark fiber for near-surface characterization and broadband seismic event detection. *Sci. Rep.* **9**, 1328. 10.1038/s41598-018-36675-8 (2019). 30718538 10.1038/s41598-018-36675-8 PMC6362068

[12] Bakku, S. Fracture characterization from seismic measurements in a borehole. In Ph.D. thesis (Massachusetts Institute of Technology, 2015).

[13] Kuvshinov, B. N. Interaction of helically wound fibre-optic cables with plane seismic waves. *Geophys. Prospect.* **64**, 671–688. 10.1111/1365-2478.12303 (2016).

[14] Masaya, S., Tani, M., Liu, Y. & Tsvankin, I. Comparison of straight and helical fibers for time-lapse elastic fwi of synthetic seafloor das data. *Lead. Edge* **43**, 757–764. 10.1190/tle43110757.1 (2024).

[15] Aki, K. & Richards, P. G. *Quantitative seismology* (University Science Books, 2002).

[16] Park, S. & Ishii, M. Near-surface compressional and shear wave speeds constrained by body-wave polarization analysis. *Geophys. J. Int.* **213**, 1559–1571. 10.1093/gji/ggy072 (2018).

[17] Abukrat, Y., Sinitsyn, P., Reshef, M. & Lellouch, A. Applications and limitations of distributed acoustic sensing in shallow seismic surveys and monitoring. *Geophysics* **88**, WC1–WC12. 10.1190/geo2022-0574.1 (2023).

[18] Katakami, S. et al. Potential of earthquake strong motion observation utilizing a linear estimation method for phase cycle skipping in distributed acoustic sensing. *J. Geophys. Res.: Solid Earth* **129**, e2023JD027327. 10.1029/2023JB027327 (2024).

[19] Crotwell, H. P., Owens, T. J. & Ritsema, J. The taup toolkit: Flexible seismic travel-time and ray-path utilities. *Seism. Res. Lett.* **70**, 154–160. 10.1785/gssrl.70.2.154 (1999).

[20] Beyreuther, M. et al. Obspy: A python toolbox for seismology. *Seism. Res. Lett.* **81**, 530–533. 10.1785/gssrl.81.3.530 (2010).

