# Supplementary material for: Comparative earthquake sensitivity of straight and triply helical fibers in surface distributed acoustic sensing
Source: Sci Rep. 2026 May 27;16:24298. doi: 10.1038/s41598-026-54482-4 (PMC13443188; doi:10.1038/s41598-026-54482-4)
Supplement: Supplementary file 1 [file 41598_2026_54482_MOESM1_ESM.pdf]

Supplementary material

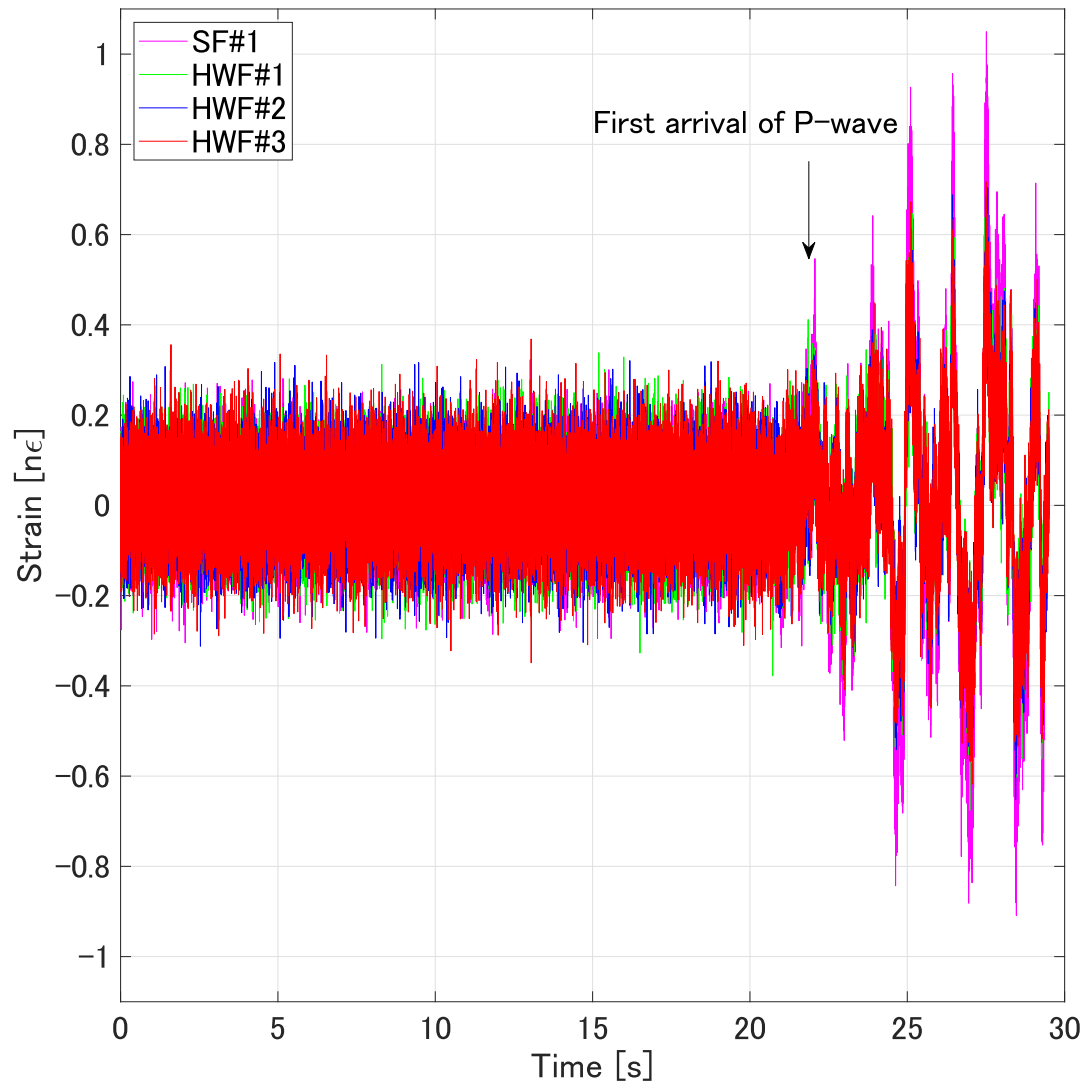

(a) 18:55:40 JST on June 11, 2023

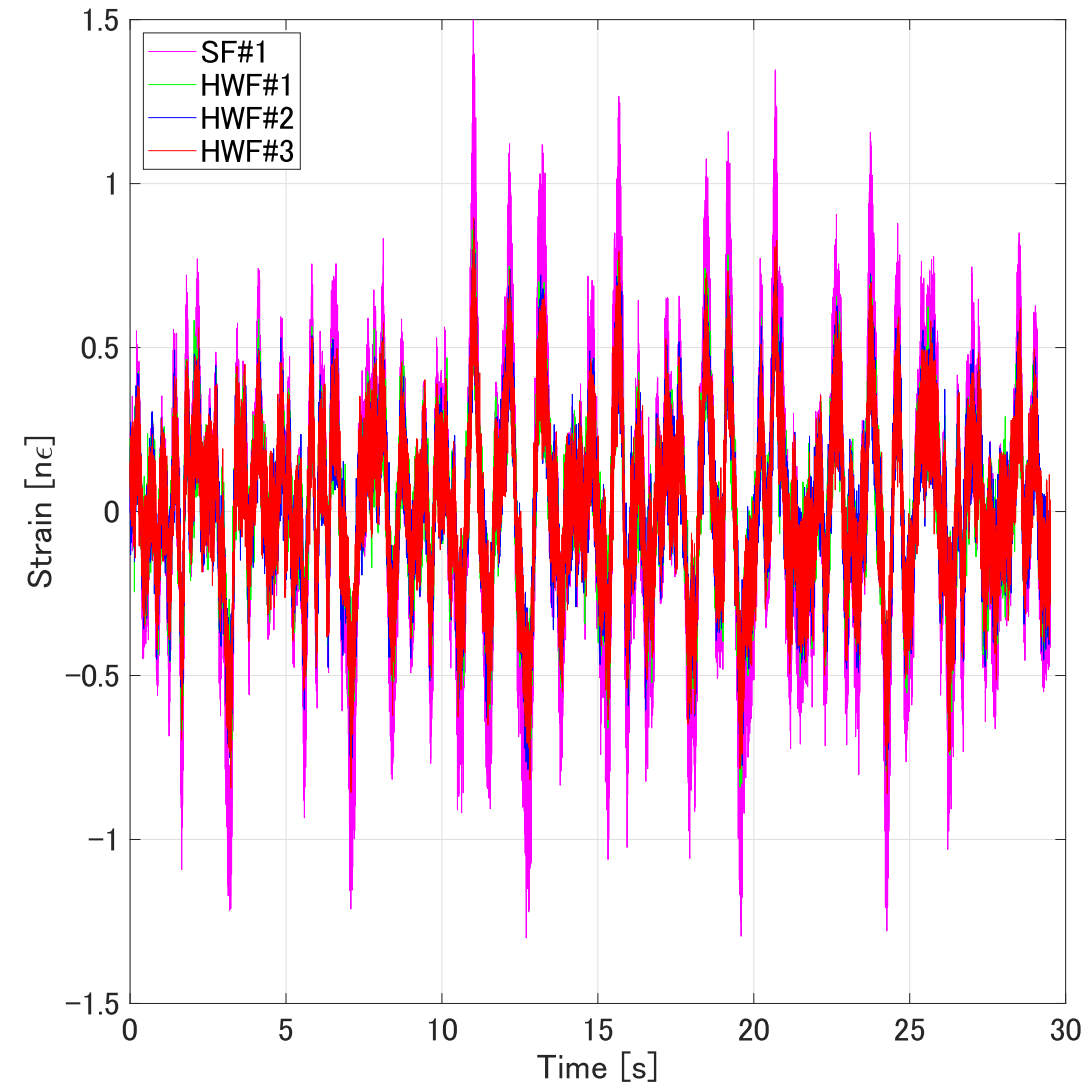

(b) 18:56:10 JST on June 11, 2023

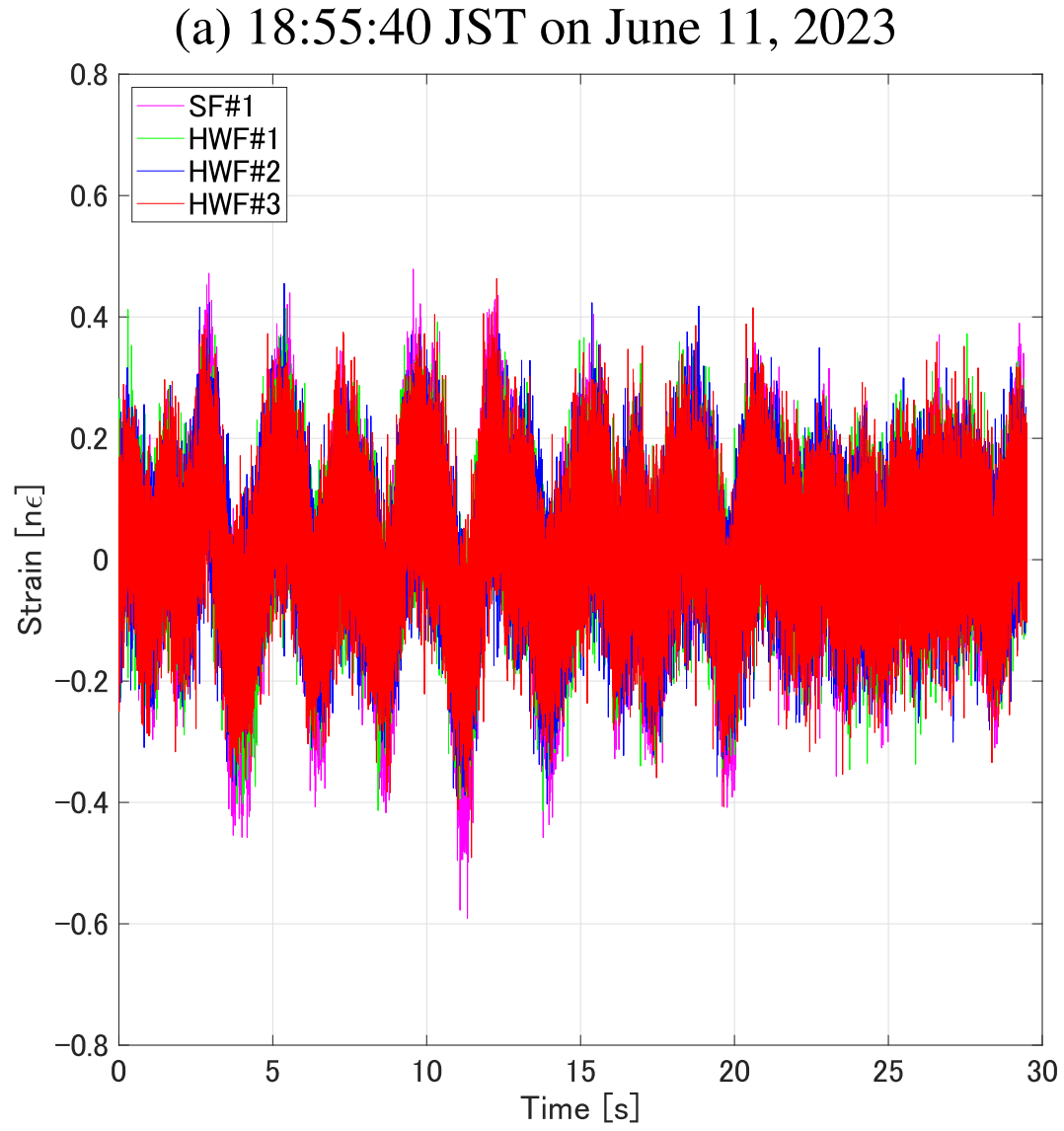

(c) 19:00:40 JST on June 11, 2023

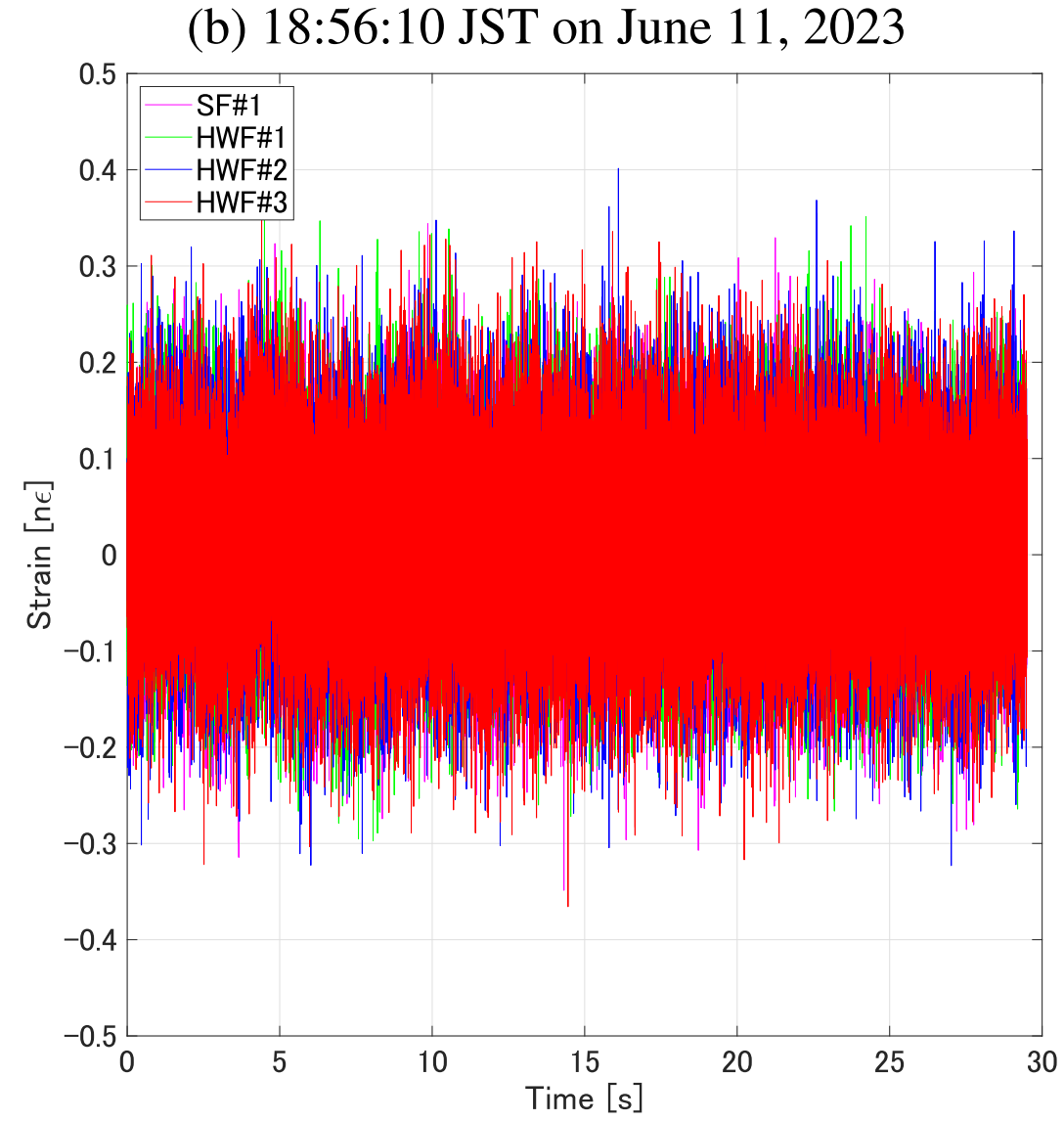

(d) 19:06:10 JST on June 11, 2023

Figure S1. Stacked seismic waveforms before and after the earthquake event at offshore Tomakomai, Japan on June 11, 2023. The 0-second point on the time axis of these graphs corresponds to the time points (a) to (d).

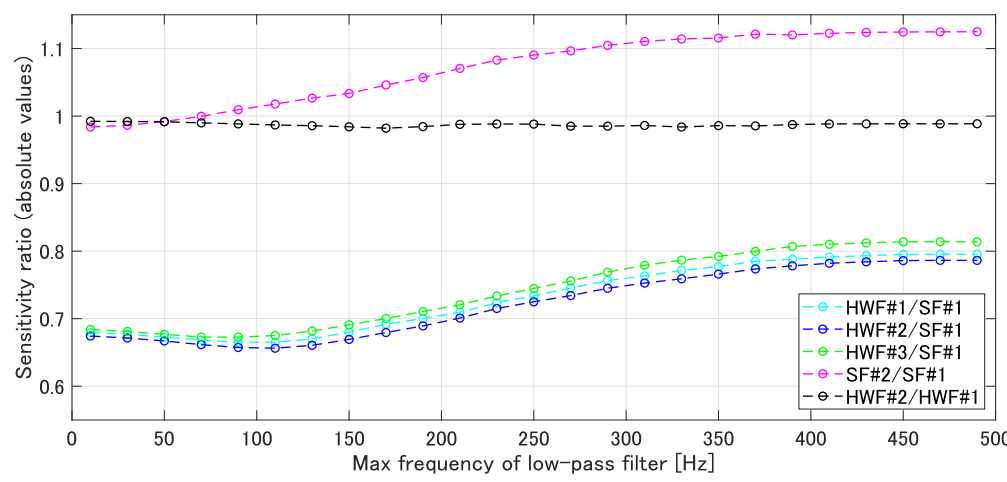

(a) 18:55:40 JST on June 11, 2023

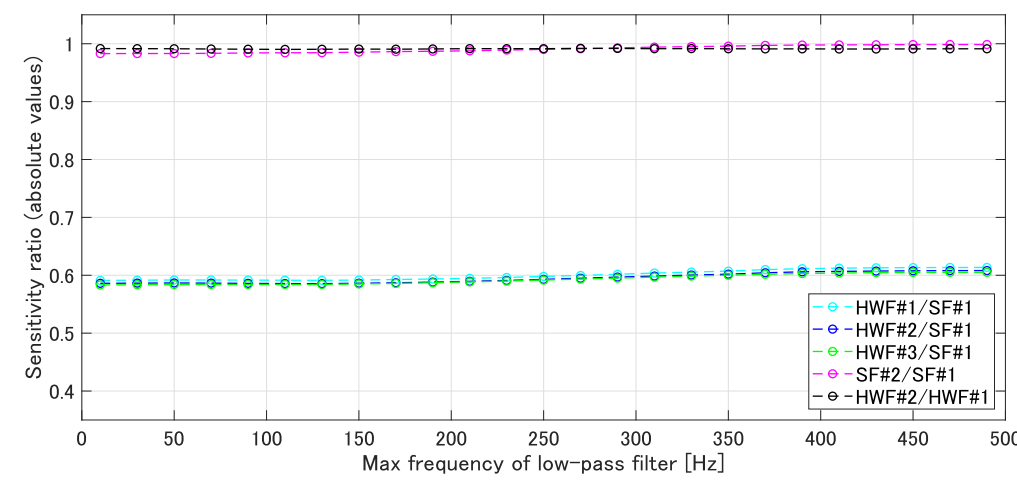

(b) 18:56:10 JST on June 11, 2023

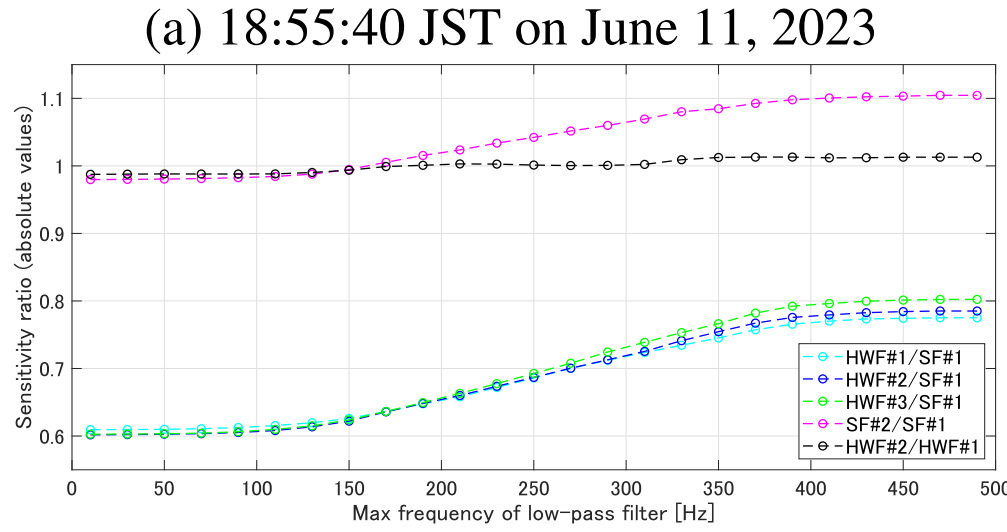

(c) 19:00:40 JST on June 11, 2023

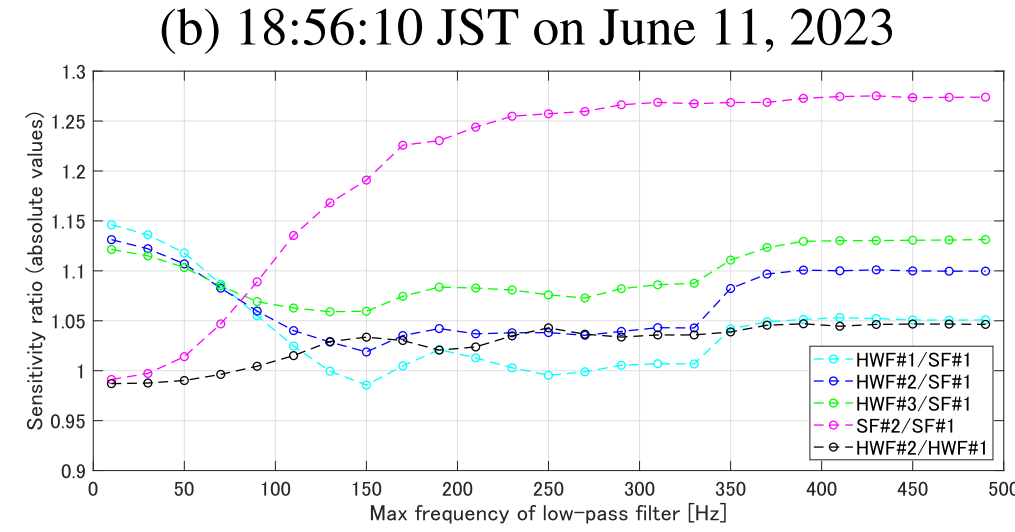

(d) 19:06:10 JST on June 11, 2023

Figure S2. Sensitivity ratio of stacked seismic waveforms before and after the earthquake event at offshore Tomakomai, Japan on June 11, 2023. These graphs represent the results of analyses using 30-second waveforms from the time points (a) to (d).

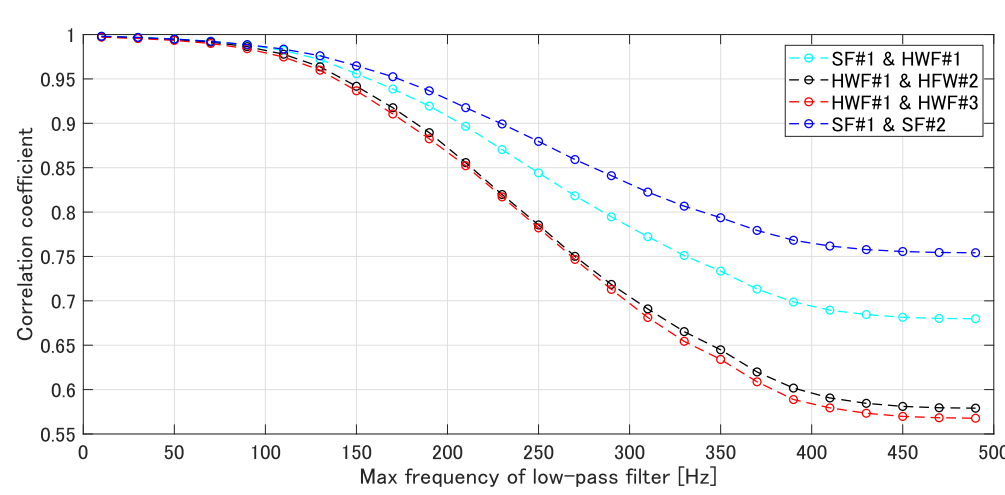

(a) 18:55:40 JST on June 11, 2023

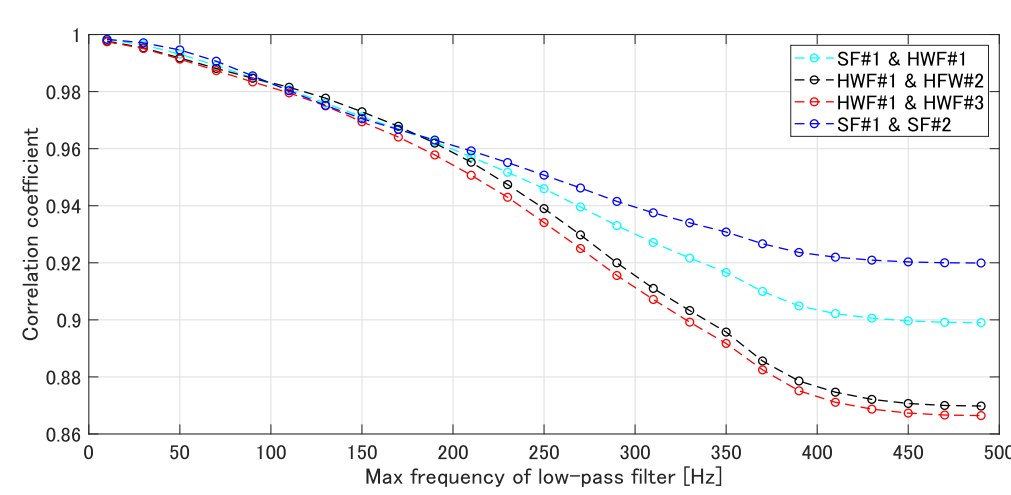

(b) 18:56:10 JST on June 11, 2023

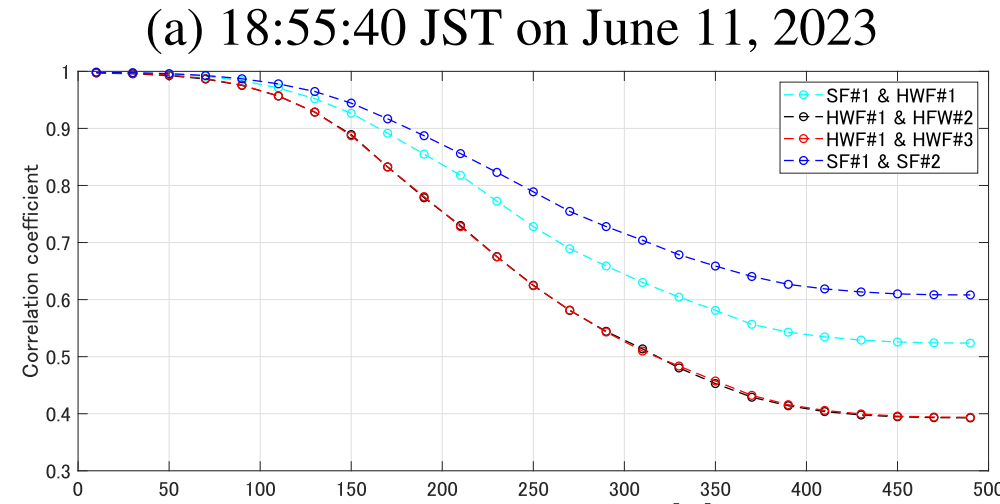

(c) 19:00:40 JST on June 11, 2023

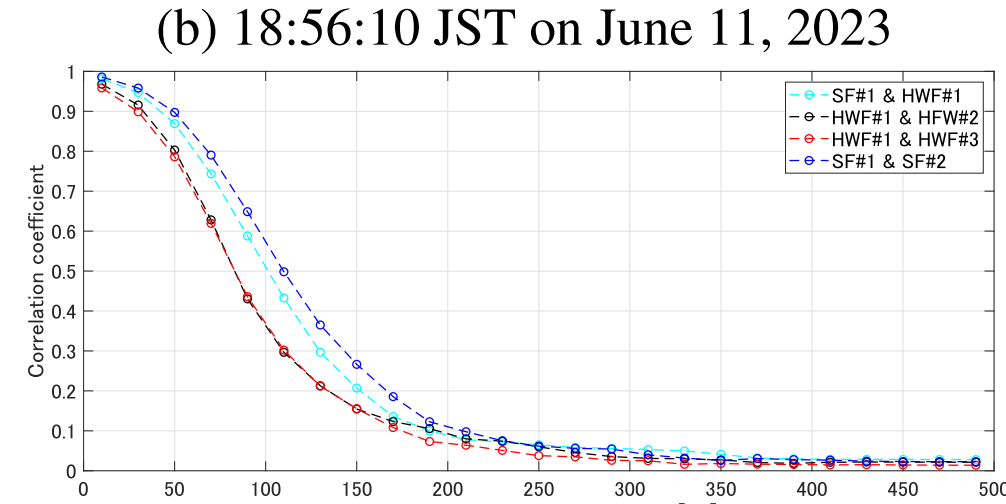

(d) 19:06:10 JST on June 11, 2023

Figure S3. Correlation coefficient of stacked seismic waveforms before and after the earthquake event at offshore Tomakomai, Japan on June 11, 2023. These graphs represent the results of analyses using 30-second waveforms from the time points (a) to (d).

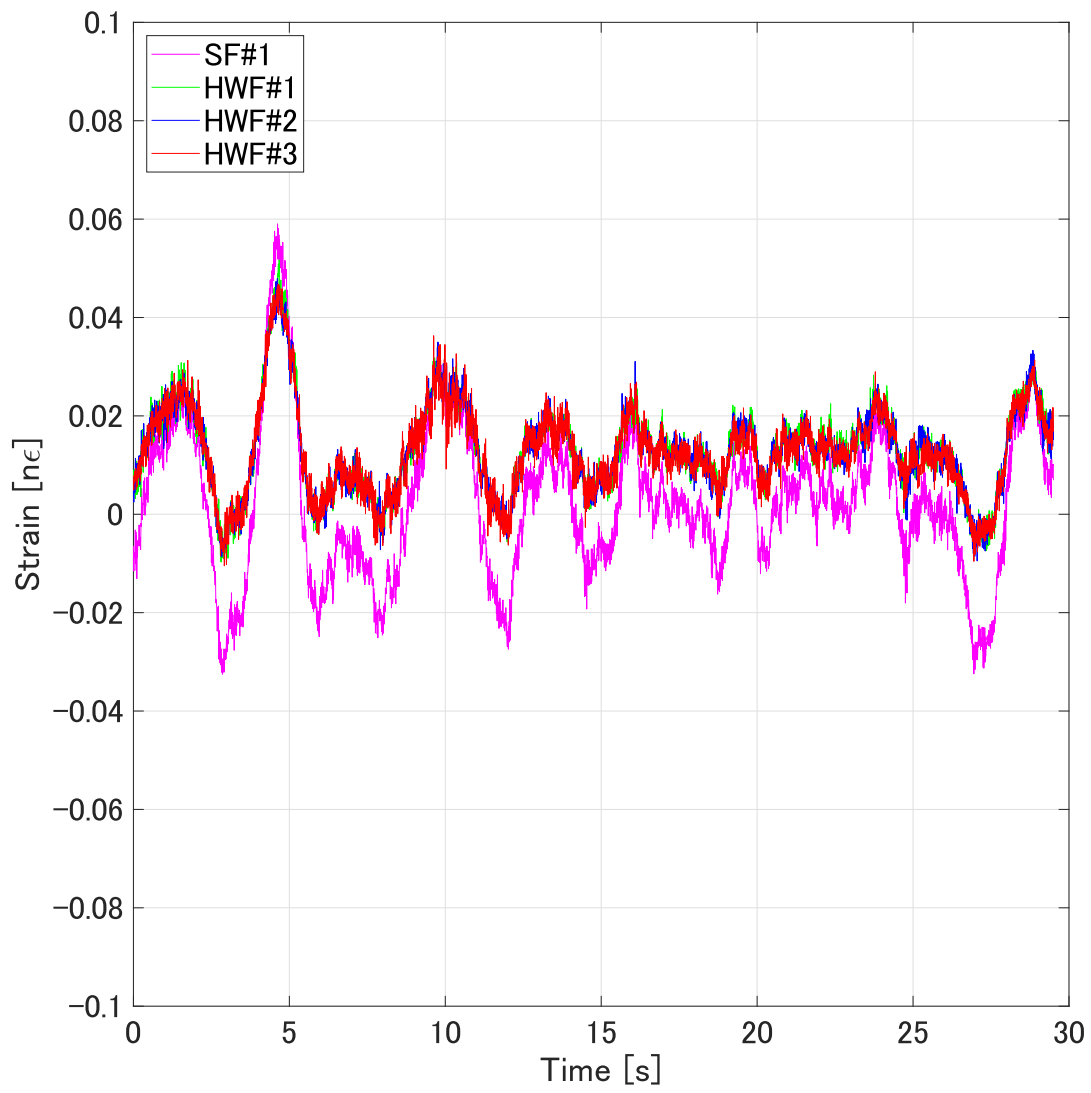

(a) 10Hz

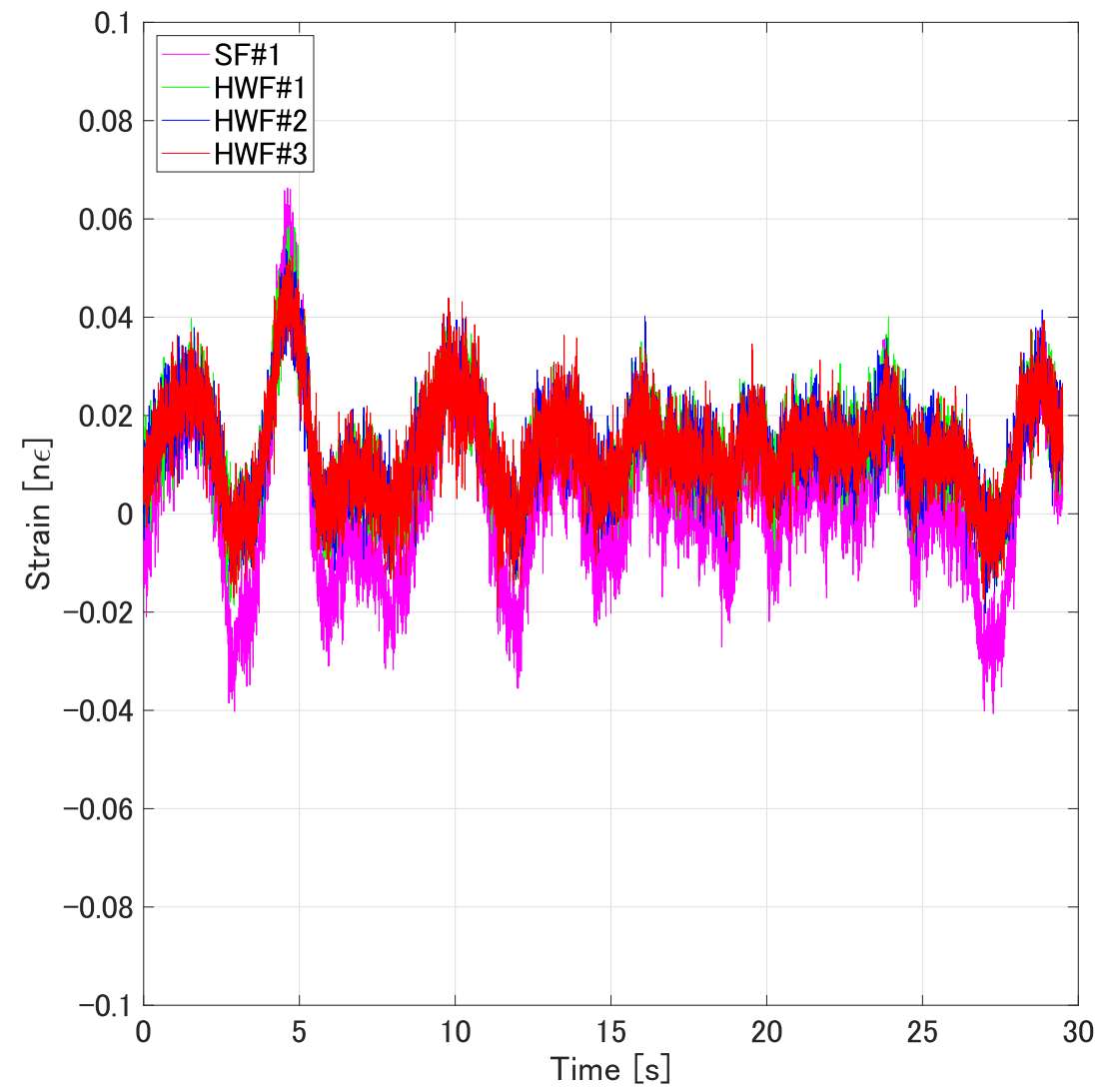

(b) 50Hz

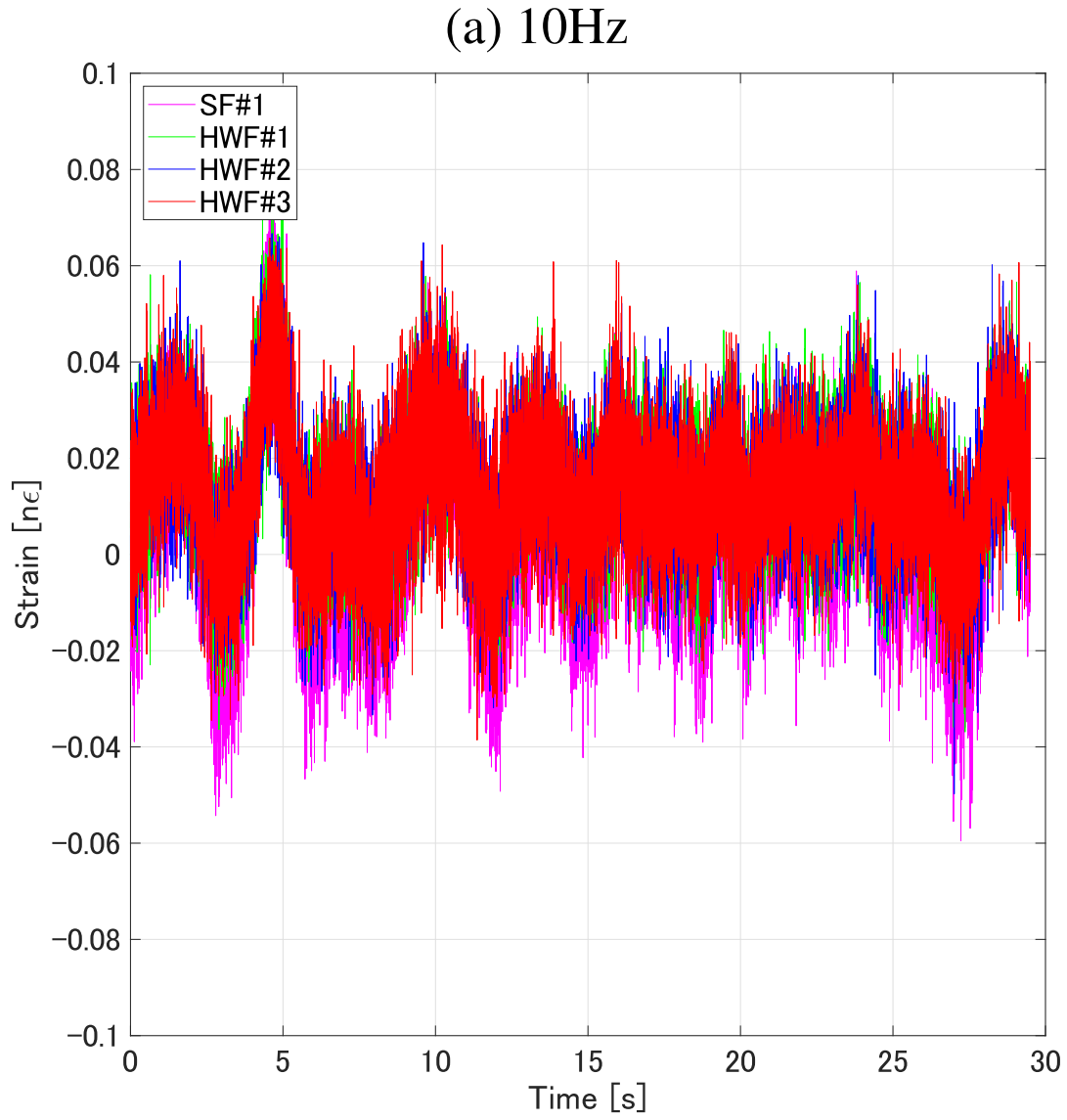

(c) 90Hz

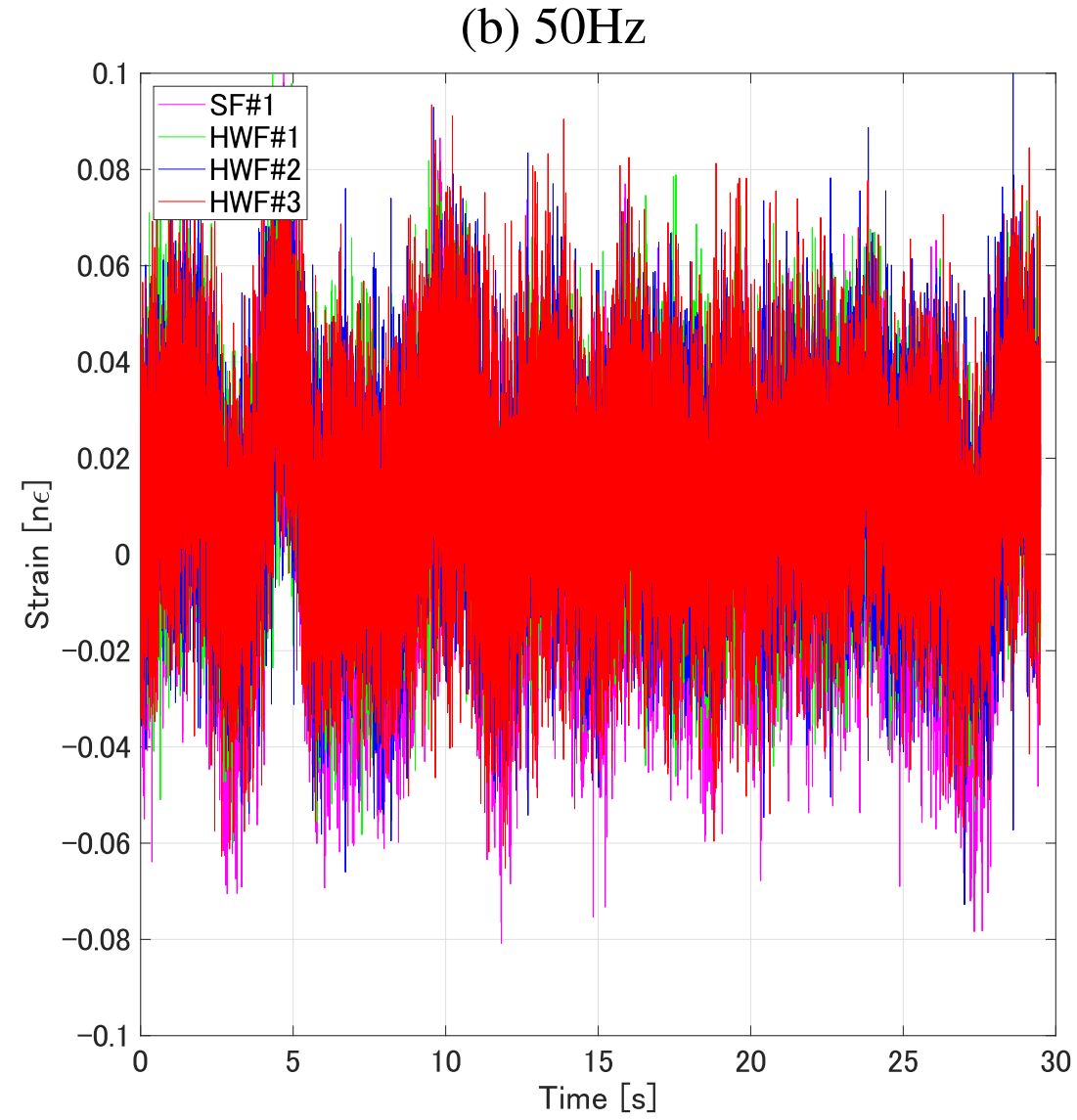

(d) 130Hz

Figure S4. Stacked seismic waveforms with the earthquake event at offshore Tomakomai after different maximum frequencies of low-pass filters. The 0-second point on the time axis of these graphs corresponds to the time point 19:06:10 JST on June 11, 2023.

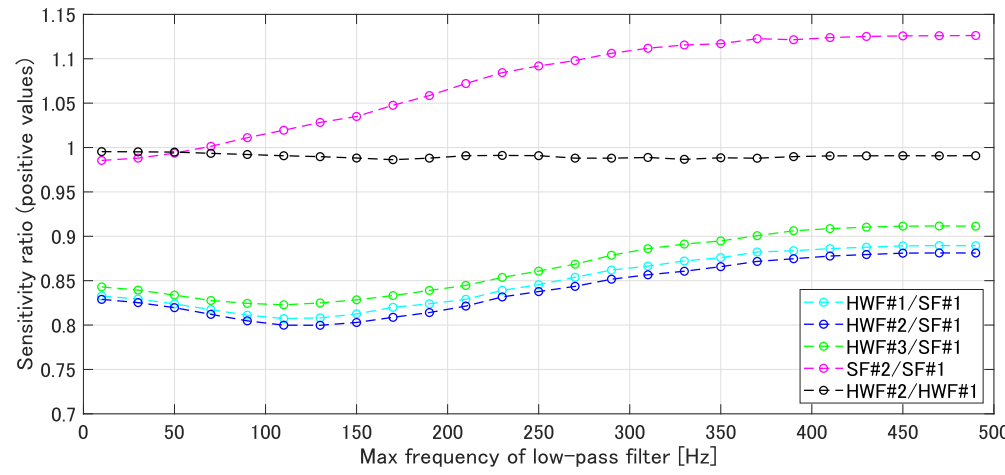

(a) 18:55:40 JST on June 11, 2023

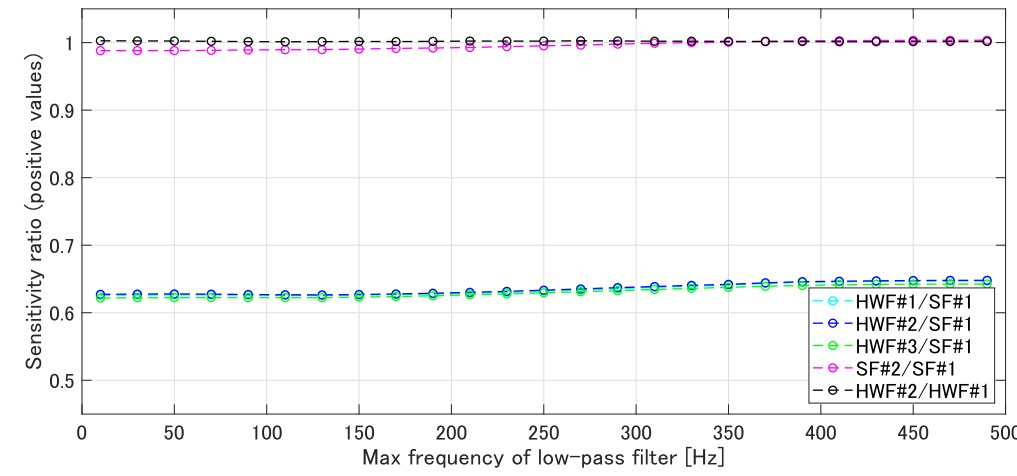

(b) 18:56:10 JST on June 11, 2023

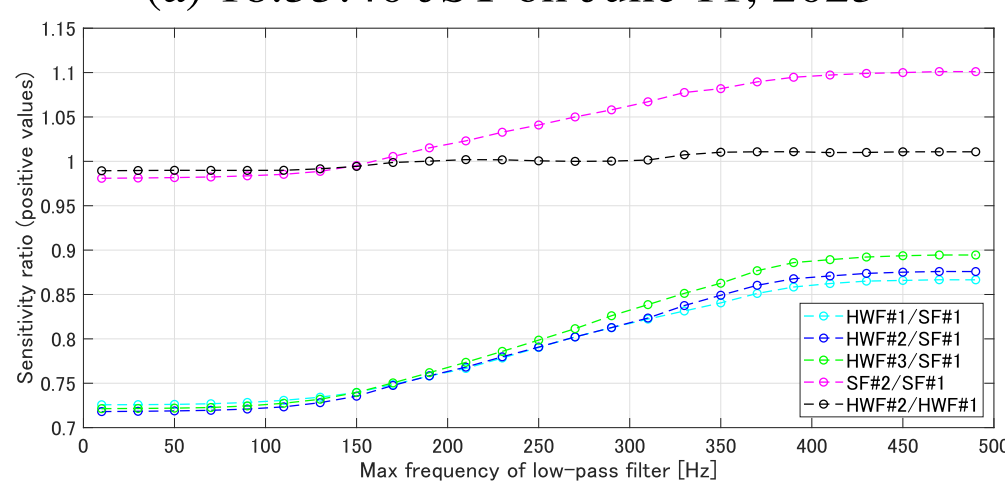

(c) 19:00:40 JST on June 11, 2023

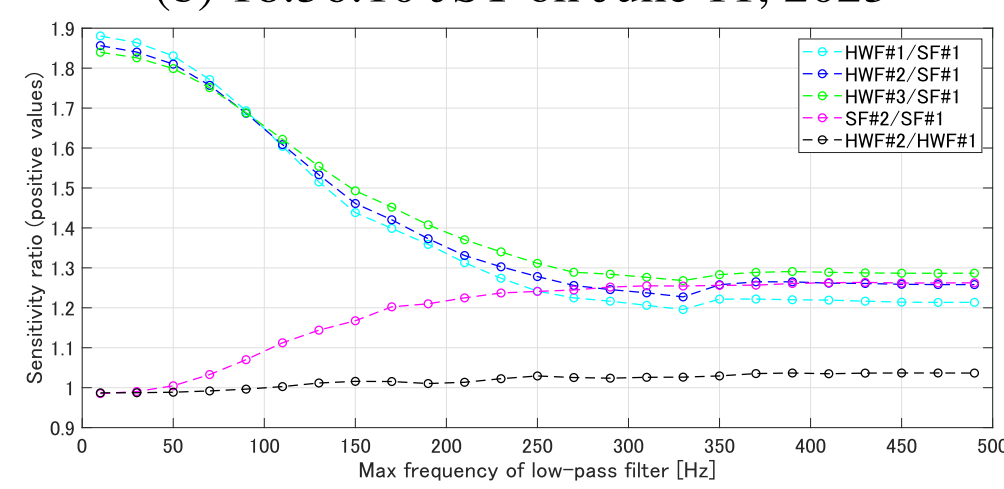

(d) 19:06:10 JST on June 11, 2023

Figure S5. Positive sensitivity ratio of stacked seismic waveforms before and after the earthquake event at offshore Tomakomai, Japan on June 11, 2023. These graphs represent the results of analyses using 30-second waveforms from the time points (a) to (d).

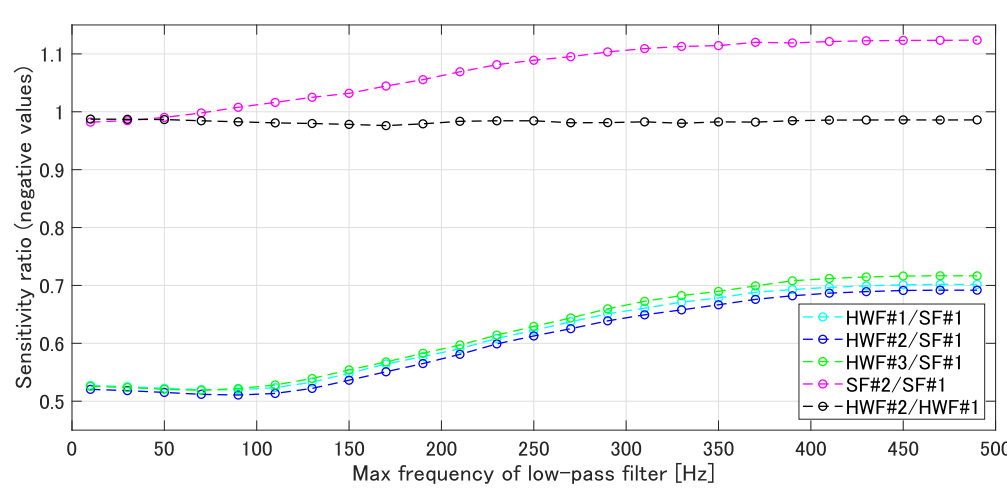

(a) 18:55:40 JST on June 11, 2023

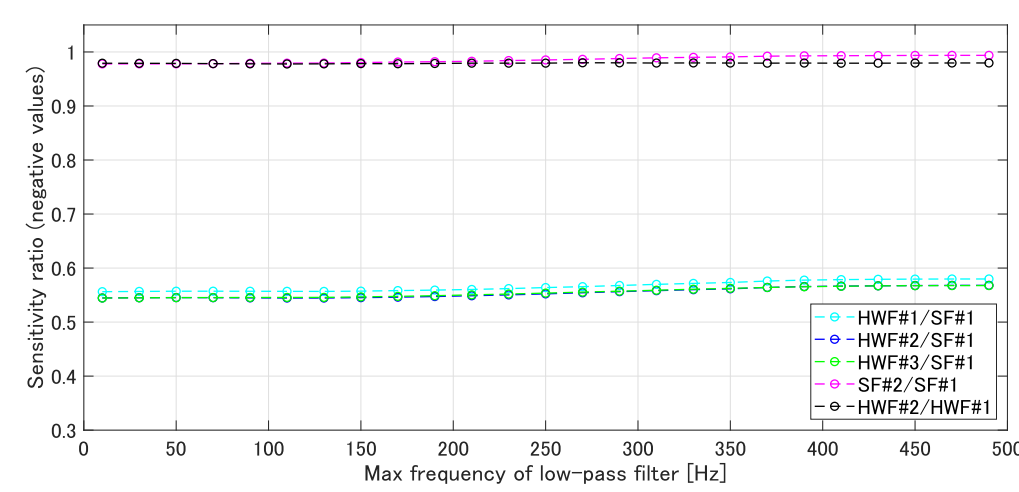

(b) 18:56:10 JST on June 11, 2023

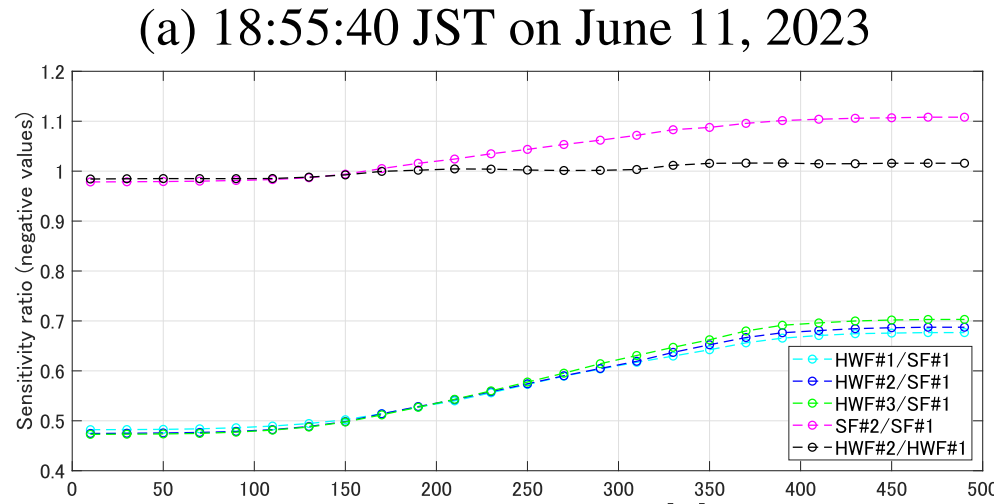

(c) 19:00:40 JST on June 11, 2023

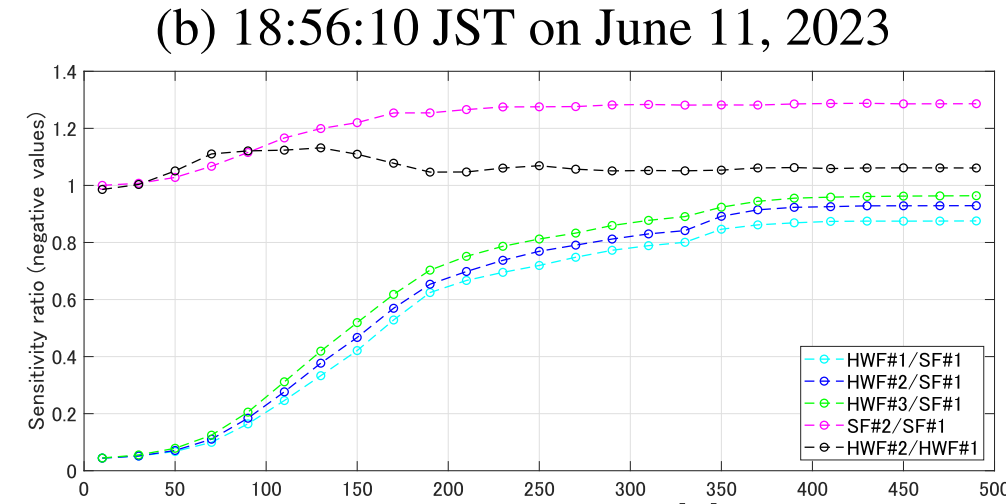

(d) 19:06:10 JST on June 11, 2023

Figure S6. Negative sensitivity ratio of stacked seismic waveforms before and after the earthquake event at offshore Tomakomai, Japan on June 11, 2023. These graphs represent the results of analyses using 30-second waveforms from the time points (a) to (d).

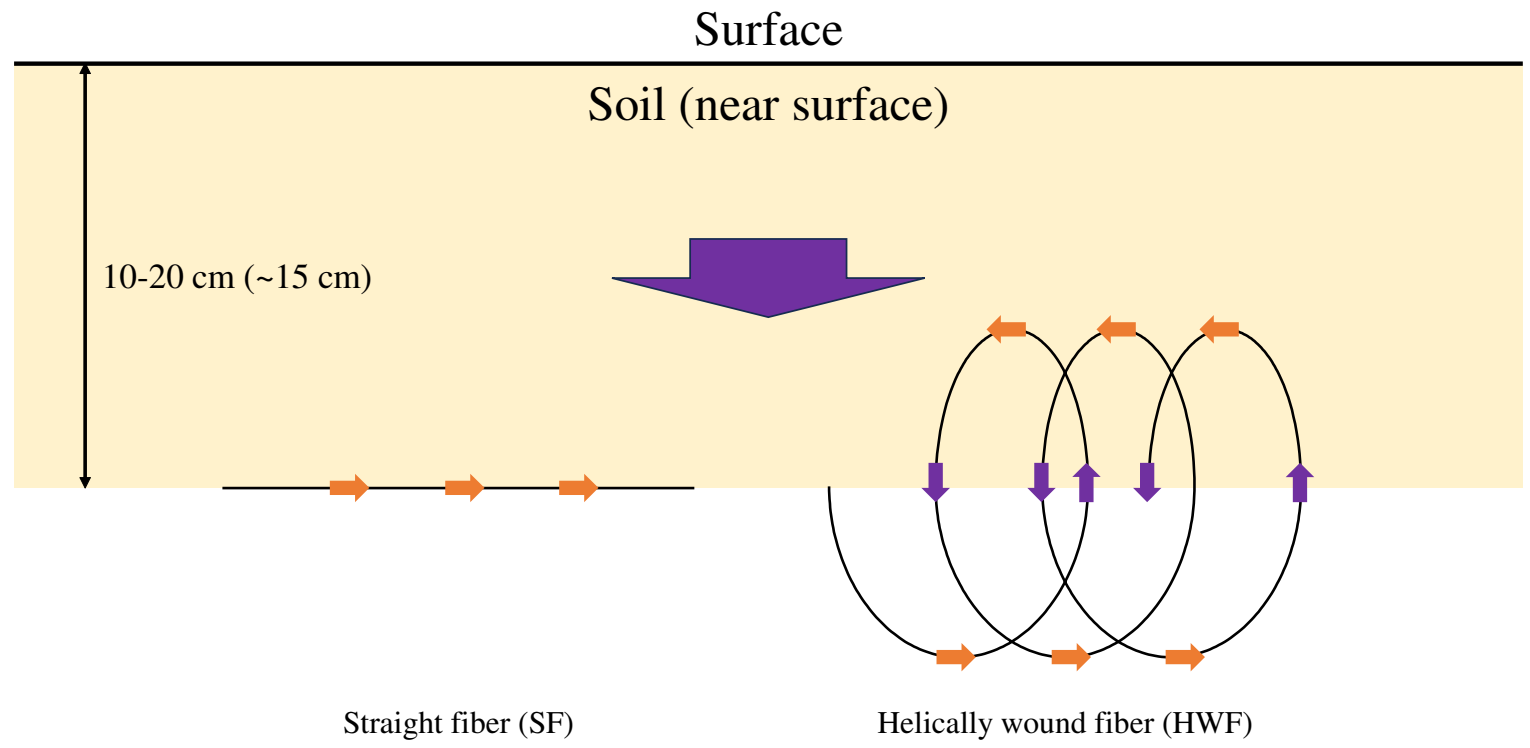

Figure S7. Schematic image showing a soil loading acting as an external force on the HWF. Note that the scale of HWF specifications and buried depth in this figure is inaccurate.

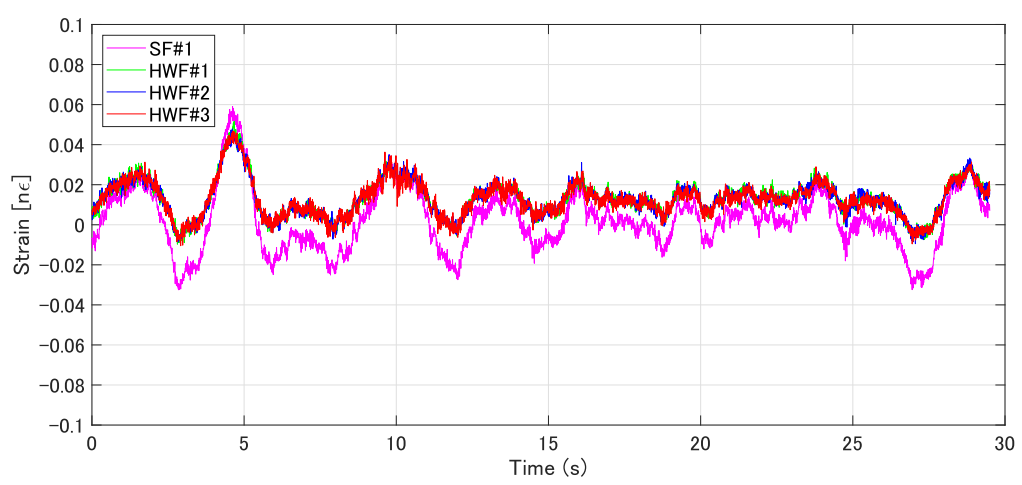

(a) Stacked seismic waveforms (before baseline shift)

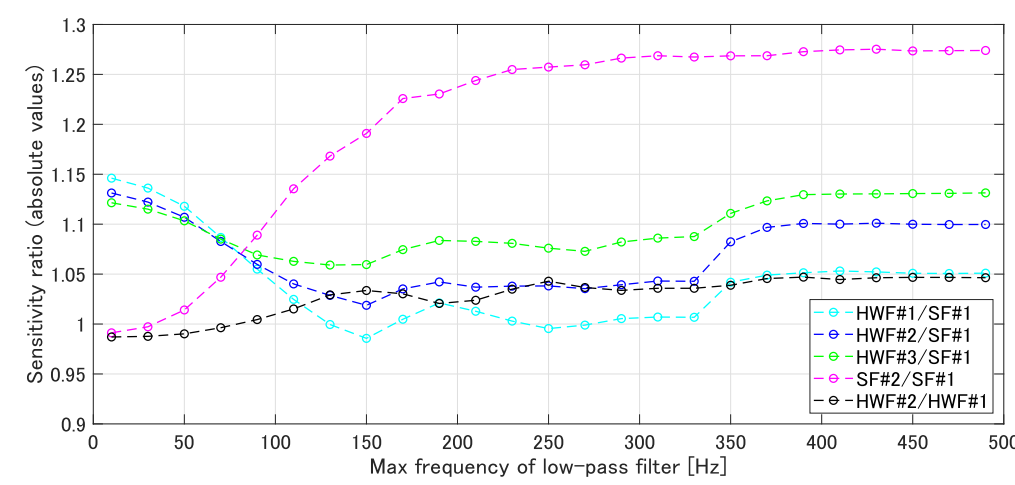

(b) Sensitivity ratio (before baseline shift)

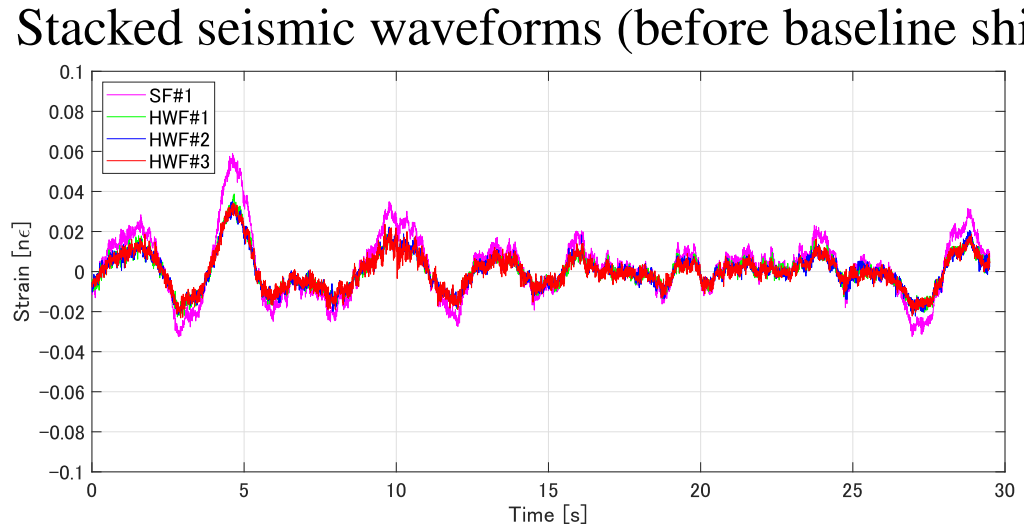

(c) Stacked seismic waveforms (after baseline shift)

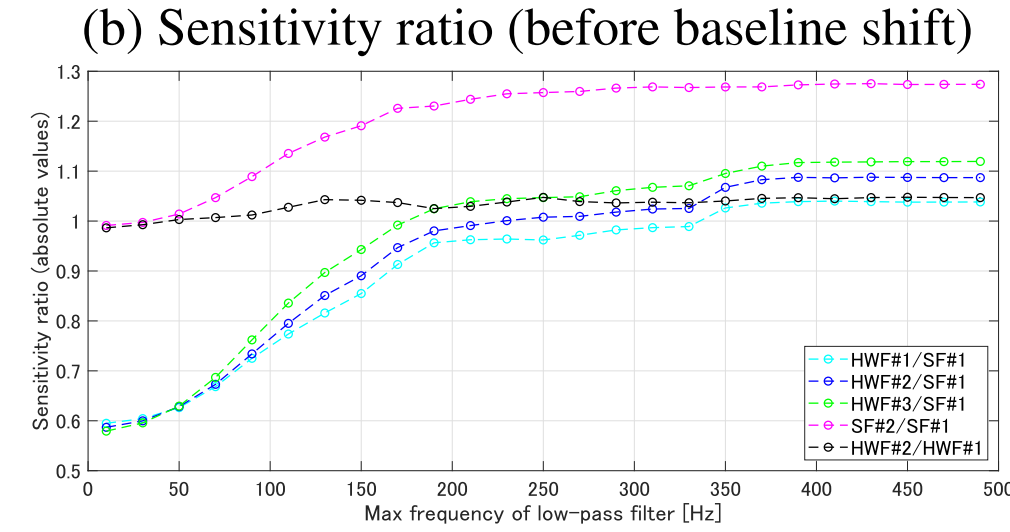

(d) Sensitivity ratio (after baseline shift)

Figure S8. Stacked seismic waveforms with the earthquake event at offshore Tomakomai after different low-cut filters of 10 Hz and sensitivity ratio of the stacked seismic waveforms. (a) and (c): The 0-second point on the time axis of these graphs corresponds to the time point 19:06:10 JST on June 11, 2023. (a)-(b): The baseline shift of the DAS data measured by HWFs is not applied. (c)-(d): The baseline shift of the DAS data measured by HWFs is applied. We subtracted positive trends equivalent to the mean values of 0.0129 s, 0.0128 s, and 0.0126 s from the waveforms measured and stacked by HWF #1, HWF #2, and HWF #3, respectively.

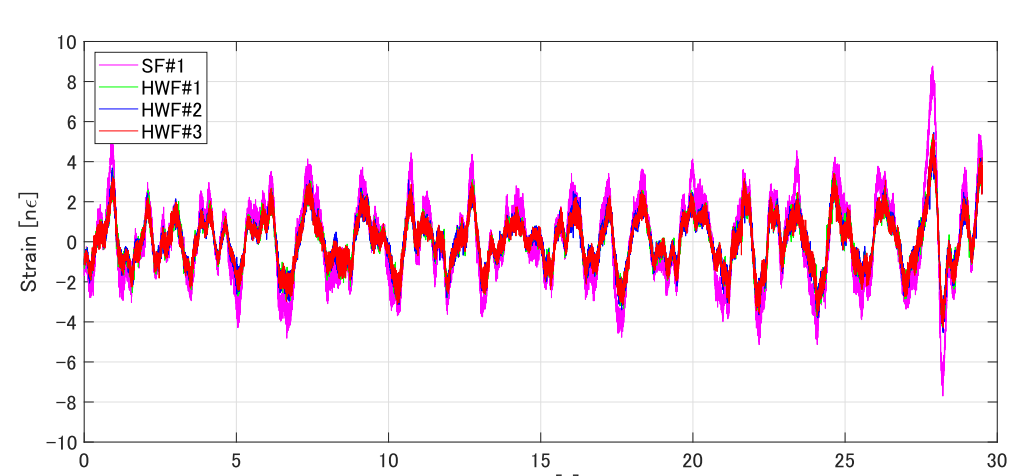

(a) Stacked seismic waveform

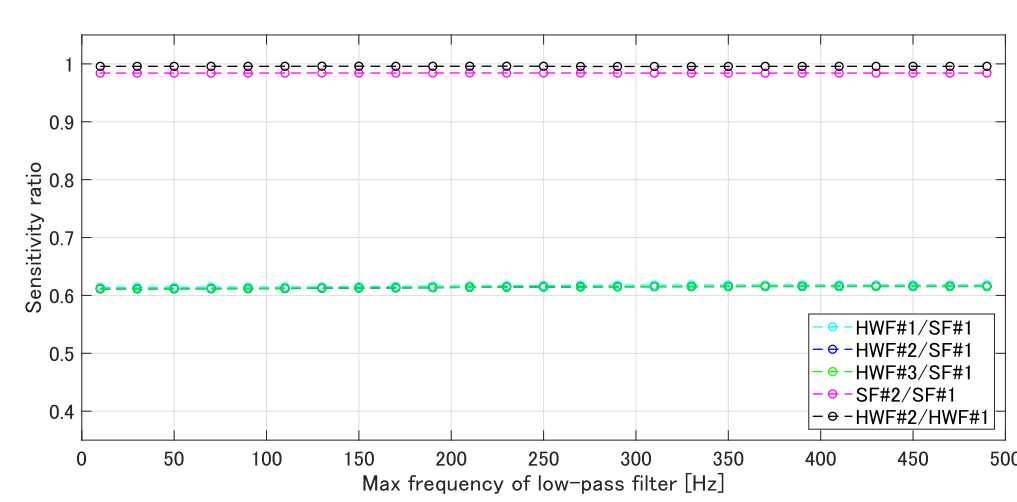

(b) Sensitivity ratio

Figure S9. Stacked seismic waveform with the earthquake event at offshore Chiba after beamforming with the stack numbers of 1909 and sensitivity ratio of the stacked seismic waveforms The 0 s time point in the waveform corresponds to 19:04:22 JST on May 26, 2023.
